# Inhibitors of the Detoxifying Enzyme of the Phytoalexin Brassinin Based on Quinoline and Isoquinoline Scaffolds

**DOI:** 10.3390/molecules22081345

**Published:** 2017-08-14

**Authors:** M. Soledade C. Pedras, Abbas Abdoli, Vijay K. Sarma-Mamillapalle

**Affiliations:** Department of Chemistry, University of Saskatchewan, 110 Science Place, Saskatoon, SK S7N 5C9, Canada; aba087@mail.usask.ca (A.A.); mas792@mail.usask.ca (V.K.S.-M.)

**Keywords:** antifungal, brassinin oxidase, camalexin, crucifer, *Leptosphaeria maculans*, paldoxin, phenylquinoline, phytoalexin detoxification

## Abstract

The detoxification of the phytoalexin brassinin to indole-3-carboxaldehyde and *S*-methyl dithiocarbamate is catalyzed by brassinin oxidase (BOLm), an inducible fungal enzyme produced by the plant pathogen *Leptosphaeria maculans*. Twenty-six substituted quinolines and isoquinolines are synthesized and evaluated for antifungal activity against *L. maculans* and inhibition of BOLm. Eleven compounds that inhibit BOLm activity are reported, of which 3-ethyl-6-phenylquinoline displays the highest inhibitory effect. In general, substituted 3-phenylquinolines show significantly higher inhibitory activities than the corresponding 2-phenylquinolines. Overall, these results indicate that the quinoline scaffold is a good lead to design paldoxins (phytoalexin detoxification inhibitors) that inhibit the detoxification of brassinin by *L. maculans*.

## 1. Introduction

**Scheme 1:**
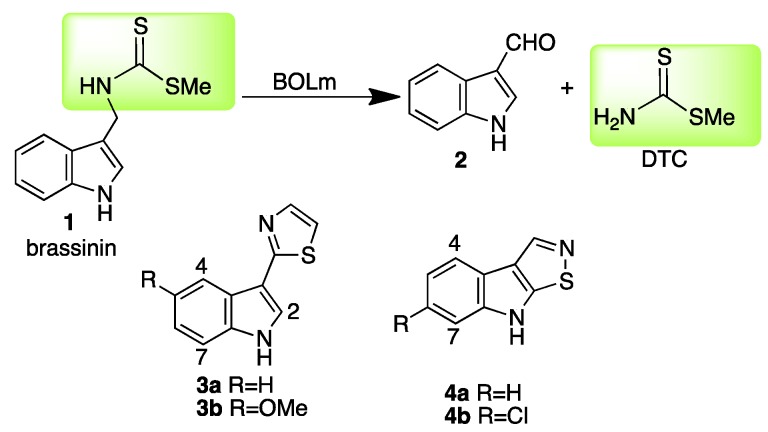
Detoxification of brassinin (**1**) by BOLm (*Leptosphaeria maculans*) and inhibitors camalexin (**3a**), 5-methoxycamalexin (**3b**), brassilexin (**4a**) and 6-chlorobrassilexin (**4b**).

The conversion of the phytoalexin brassinin (**1**) to indole-3-carboxaldehyde (**2**) is catalyzed by brassinin oxidase (BOLm), the only enzyme currently known to mediate the transformation of a dithiocarbamate to an aldehyde [1,2]. This transformation is a detoxification process that eliminates the toxophore *S*-methyl dithiocarbamate of brassinin to afford non-toxic products (Scheme 1).

BOLm is an inducible phytoalexin detoxifying enzyme produced by a fungal pathogen of crucifer crops (*Leptosphaeria maculans* (Desm.) Ces. et de Not., asexual stage *Phoma lingam* (Tode ex Fr.) Desm.) that causes major epidemics worldwide. Brassinin (**1**) is a cruciferous phytoalexin produced by plants of the family Brassicaceae (common name crucifer), which include globally cultivated crops belonging to the *Brassica* genus [3]. Brassica crops are of enormous importance worldwide as sources of oil, food, feed, and fuel. Brassinin (**1**) is an important phytoalexin because it functions as antimicrobial plant defense and as biosynthetic precursor of several phytoalexins; depletion of brassinin (**1**) through detoxification is a pathogen’s strategy to weaken the defense system of brassicas [3]. In principle, inhibition of such a detoxification transformation could allow brassinin (**1**) to build up in plant cells and stop pathogen growth.

As part of a research program to devise sustainable methods to protect plants against fungal infections, we are particularly interested in the development of paldoxins, i.e., phytoalexin detoxification inhibitors [4]. Paldoxins of BOLm [5,6] are being considered as potential crop protectants having a specific mechanism of action, the inhibition of brassinin detoxification by *L. maculans* [7]. The attraction of this approach lies in the possibility of exploiting paldoxins as selective fungal enzyme inhibitors. It is anticipated that such selective inhibitors will display lower toxicity levels to the encompassing ecosystem and thus are less likely to have a negative environmental impact than conventional fungicides.

Particularly because BOLm has not been expressed in heterologous systems and only relatively small quantities can be obtained using classic chromatographic techniques, in depth structural studies have not been carried out. Consequently, since the tertiary structure of BOLm remains unknown and no relevant model systems have been reported, the design of inhibitors of BOLm is an ongoing challenge. Preliminary screening of a library of more than 80 synthetic brassinin analogues and isosteres, designed by replacement of the dithiocarbamate group of **1** with carbamate, dithiocarbonate, urea, thiourea, sulfamide, sulfonamide, dithiocarbazate, amide, and ester functionalities, plus replacement of the indolyl moiety with naphthalenyl and phenyl, did not identify BOLm inhibitors [8]. Unexpectedly, among several natural products, the phytoalexins camalexin (**3a**) [1] and brassilexin (**4a**) [5] were found to inhibit BOLm. Upon optimization of both lead structures, inhibitors of BOLm more potent than the parent compounds were obtained, 5-methoxycamalexin (**3b**) and 6-chlorobrassilexin (**4b**) became the best competitive inhibitors of BOLm [7]. However, both **3b** and **4b** displayed stronger antifungal activity, a characteristic less desirable in potential paldoxins due to potential toxicity to the plant and surrounding living organisms. Hence, it is of interest to develop new scaffolds containing different heterocyclic systems to establish structural correlations among BOLm inhibitors and their antifungal activities against *L. maculans*. Herein, the inhibition of BOLm and the antifungal activities of a new series of compounds having quinoline and isoquinoline skeletons are reported.

## 2. Results and Discussion

### 2.1. Design and Synthesis of Potential Inhibitors of BOLm

The most significant inhibitors of BOLm discovered to date are derived from indolyl containing scaffolds, namely 5-methoxycamalexin (**3b**) and 6-chlorobrassilexin (**4b**) [7]. Quinolines are monoazanaphthalenes that can be formally considered structural hybrids of indolyl and naphthalenyl skeletons. For this reason, quinolines, and their structural isomers isoquinolines, are heterocyclic scaffolds of interest to us as potential inhibitors of BOLm. A few quinoline derivatives currently used as commercial agricultural fungicides include quinoxyfen and tebufloquin, which are known to affect signal transduction pathways and the respiratory system, respectively [9], and tubulin polymerization [10,11].
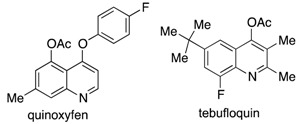


**Figure 1:**
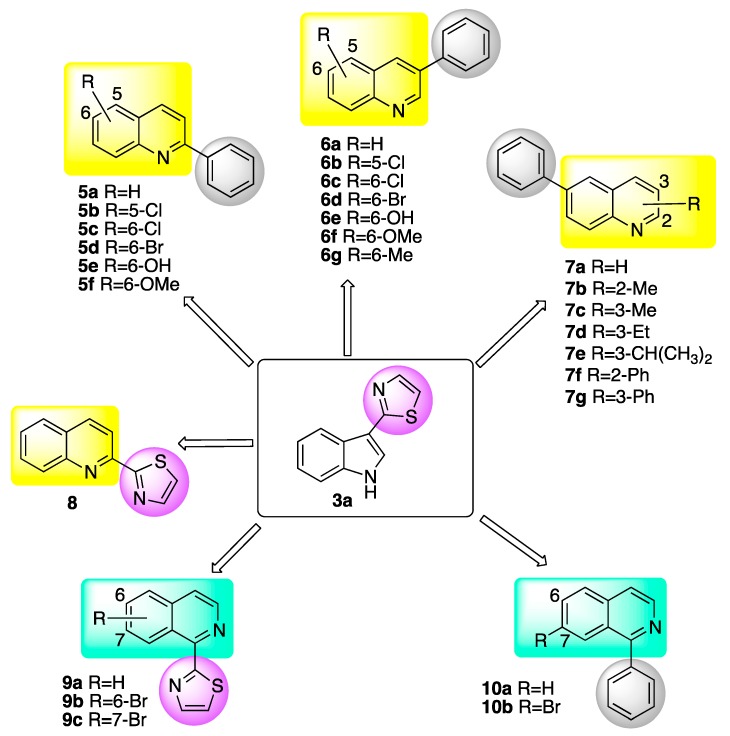
Quinolines **5a**–**8** and isoquinolines **9a**–**10b** evaluated for inhibition of BOLm.

Considering the above framework, the development of quinolines with a new mechanism of action that targets specific fungal pathogens and offers selectivity is of great interest for crop protection. Figure 1 shows the substituted quinolines and isoquinolines designed and synthesized for this purpose.

Syntheses of brassinin (**1**) [8], camalexin (**3a**) [12], and isoquinolines **8** [13] and **9a** [13] were carried out as previously reported. Chemical syntheses of new quinolines **6e**, **7c**–**7e**, **7g**, improved syntheses of known quinolines **5a**–**5f**, **6a**–**6d**, **6f**, **7a**, **7b** and **7f** and syntheses of new isoquinolines **9b**, **9c** and **10b** were carried out as described below.

#### 2.1.1. Quinolines

Quinolines can be synthesized using a number of classical methods including the Friedländer, Skraup, Combes, and Doebner–von Miller syntheses from anilines and carbonyl compounds, as well as metal-catalyzed dehydrogenative cyclization and other “greener” methods [14,15,16,17]. In the current work, quinolines **5a**–**5f**, **6a**–**6f** and **7a**–**7g** were synthesized using the Friedländer method by condensation of the corresponding 2-aminobenzaldehydes, prepared from substituted 2-nitrobenzaldehydes [18], with aldehydes or ketones. In short, 2-nitrobenzaldehydes **11a**–**11g** were reduced with iron powder to the corresponding amines by refluxing in EtOH/HCl for 40 min [19]. Aldehydes **12b**–**12d** and **12f**–**12h** or ketones **12a**, **12e** and **12i** and KOH (1.2 eq.) were added to the reaction mixture that was kept at RT for 30 min, followed by heating under reflux for 3 h, to afford the corresponding quinolines **5a**–**5f**, **6a**–**6f** and **7a**–**7g** in reasonable yields (Table 1).

#### 2.1.2. Isoquinolines

**Scheme 2:**
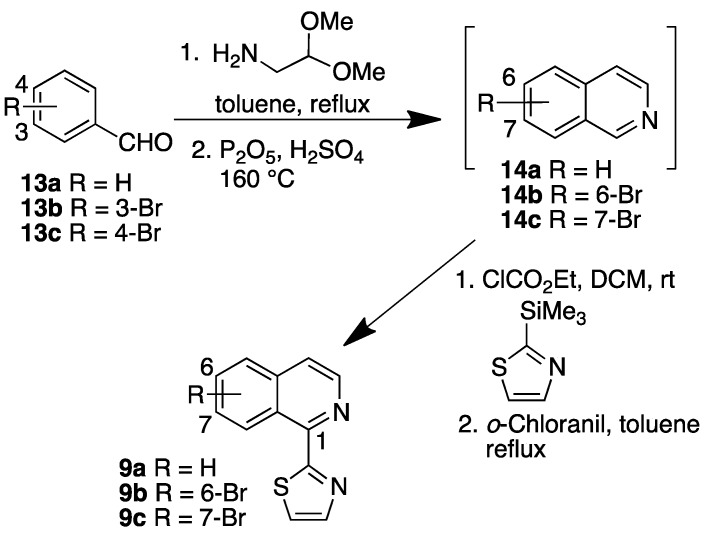
Synthesis of isoquinolines **9a**–**9c**.

The best known classical approaches to isoquinoline scaffolds are the Bischler–Napieralski synthesis, in which a β-arylethylamide is converted into a 3,4-dihydroisoquinoline derivative, the Pictet–Spengler reaction that involves an acid-catalyzed intramolecular cyclization, and the Pomeranz–Fritsch reaction that uses benzylamino acetals [17]. In addition, a large variety of methods that require less harsh conditions are available. In this work, substituted isoquinolines were synthesized from their corresponding bromoisoquinolines **14a**–**14c**, which were prepared by a modification of the Pomeranz–Fritsch reaction [20,21]. Benzaldehydes **13a**–**13c** were treated with aminoacetaldehyde dimethylacetal (Dean–Stark conditions) followed by cyclization in the presence of H_2_SO_4_/P_2_O_5_ to yield isoquinolines **14a**–**14c**. Thiazole substituted isoquinolines **9a**–**9c** were prepared from isoquinolines **14a**–**14c** by treatment with ethyl chloroformate followed by 2-trimethylsilylthiazole and deprotection using *o-*chloranil (Scheme 2).

**Scheme 3:**
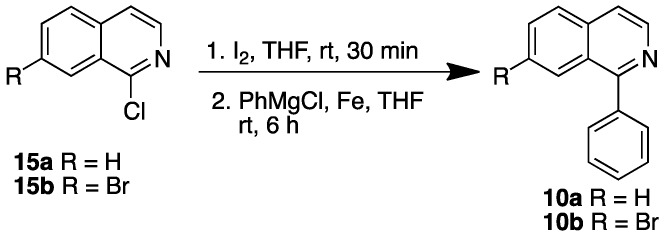
Synthesis of isoquinolines **10a** and **10b**.

Syntheses of 1-phenylisoquinolines **10a** and **10b** were carried out by modifying a reported procedure [22]; 1-chloroisoquinolines **15a** and **15b** were treated with iodine followed by PhMgCl and Fe powder in THF (Scheme 3). Unlike in the reported procedure, none of the reactions proceeded in the absence of iodine.

### 2.2. Antifungal Activity

The phytoalexins brassinin (**1**) and camalexin (**3a**) were used as reference compounds to compare the antifungal activities of synthetic compounds with those of known phytoalexins. The antifungal activity of **1**, **3a**, quinolines **5a**–**8** and isoquinolines **9a**–**10b** against *L. maculans* was determined employing the mycelial growth inhibition assay [23] described in Materials and Methods. The mycelial growth of each plate was measured after incubation for five days and the results were statistically analyzed (Table 2, results of six independent experiments conducted in triplicate). In general, quinoline derivatives (0.50 mM) showed weaker antifungal activity than camalexin (**3a**), except for 3-phenylquinoline (**6a**) and 6-methoxy-3-phenylquinoline (**6f**), whereas 5-chloro-3-phenylquinoline (**6b**) displayed the lowest growth inhibitory activity. 3-Phenylquinoline (**6a**) showed stronger antifungal activity than its structural isomer 6-phenylquinoline (**7a**), whereas structural isomers 6-methyl-3-phenylquinoline (**6g**) and 3-methyl-6-phenylquinoline (**7c**) caused similar mycelial growth inhibition. Interestingly 3,6-diphenylquinoline (**7g**) was not growth inhibitory and 1-(2-thiazolyl)isoquinoline (**9a**) was the most inhibitory of all tested compounds.

### 2.3. Inhibition of Brassinin Oxidase Activity

Cell-free protein extracts of mycelia of *L. maculans* containing BOLm activity induced by 3-phenylindole were employed (prepared as described in Section 3.4) [1] to test the potential paldoxin activity of quinolines **5a**–**8** and isoquinolines **9a**–**10b**. Compounds were evaluated at concentrations (0.10 and 0.30 mM) close to the concentration of substrate required for half-maximal activity (*K_M_* = 0.15 mM), in the presence of brassinin (**1**, 0.10 mM) and phenazine methosulfate (PMS) as the electron acceptor cofactor (BOLm accepts a wide range of cofactors: PMS, small quinones or flavin mononucleotide, FMN) [1]. To compare the inhibitory activity of all new compounds with those previously reported [7], camalexin (**3a**) was used as a standard due to its established BOLm inhibitory activity (ca. 53% at 0.30 mM) and chemical stability [6]. Results of these assays are presented in Table 3.

It is remarkable that of the 26 compounds tested, 11 inhibited BOLm activity, but none of the halogenated quinolines or isoquinolines affected the activity of BOLm (Table 3). Except for 2-methyl-6-phenyl-quinoline (**7b**), all substituted 6-phenylquinolines inhibited BOLm activity. 3-Ethyl-6-phenylquinoline (**7d**) showed the highest inhibitory effect (ca. 64% at 0.30 mM), somewhat stronger than that of camalexin (**3a**), followed by 6-methoxy-3-phenylquinoline (**6f**) (ca. 40% at 0.30 mM), whereas 6-hydroxy-3-phenylquinoline (**6e**), 6-phenylquinoline (**7a**), 3-methyl-6-phenylquinoline (**7c**), and 3-isopropyl-6-phenylquinoline (**7e**) showed comparable inhibitory effects. Substituted 3-phenylquinolines showed significantly higher inhibitory activities than the corresponding 2-phenylquinolines. The number of structures evaluated is still insufficient to make general conclusions regarding the structure–activity correlations of quinolines and isoquinolines, compounds with substituents at C-4, C-7, and C-8 ought to be considered. Nonetheless, it is apparent and relevant to note that halogenated compounds did not show inhibitory activity against BOLm, contrary to results previously obtained with camalexin (**3a**) and brassilexin (**4a**) derivatives in which halogen-substituted compounds (e.g., **4b**) were somewhat more inhibitory of BOLm activity [7].

## 3. Materials and Methods

### 3.1. General

Chemicals were purchased from Alfa Aesar, Ward Hill, MA or Sigma-Aldrich Canada Ltd., Oakville, ON, Canada; solvents were HPLC grade and used as such. Flash column chromatography (FCC) was carried out using silica gel grade 60, mesh size 230–400 Å or WP C18 prepscale bulk packing 275 Å (J.T. Baker, NJ, USA). Organic extracts were dried over Na_2_SO_4_ and concentrated using a rotary evaporator.

NMR spectra were recorded on Bruker 500 Avance spectrometers (Bruker Corporation, Billerica, MA, USA), for ^1^H, 500.3 MHz and for ^13^C, 125.8 MHz; chemical shifts (δ) are reported in parts per million (ppm) relative to TMS; spectra were calibrated using the solvent peaks; spin coupling constants (*J*) are reported to the nearest 0.5 Hz. FTIR data were recorded on a Bio-Rad FTS-40 spectrometer(Bio-Rad Laboratories, Inc., Hercules, CA, USA) and spectra were measured by the diffuse reflectance method on samples dispersed in KBr. HREI-MS were obtained on a VG 70 SE mass spectrometer(Waters Technologies Corporation, Milford, MA, USA) employing a solids probe or on a Jeol AccuToF GCv 4G mass spectrometer (Jeol USA, Peabody, MA, USA) [field desorption (FD)] by direct insertion.

HPLC analysis was carried out with Agilent high performance liquid chromatographs equipped with quaternary pump, automatic injector, and photodiode array detector (DAD, wavelength range 190–600 nm), degasser, and a column Eclipse XDB-C18 (Agilent Technologies, Santa Clara, CA, USA) (5 µm particle size silica, 4.6 mm i.d. × 150 mm), having an in-line filter, using methods: A, mobile phase 80% H_2_O—20% CH_3_CN for 45.0 min, linear gradient, at a flow rate of 0.40 mL/min; B, mobile phase 50% H_2_O—50% MeOH to 100% MeOH for 25.0 min, linear gradient, at a flow rate 0.75 mL/min.

### 3.2. Synthesis

Syntheses of brassinin (**1**) [8], camalexin (**3a**) [12], and isoquinolines **8** [13] and **9a** [13] were carried out as previously reported. Satisfactory spectroscopic data identical to those previously reported were obtained for all known compounds.

#### 3.2.1. Phenylquinolines **5a**–**7g**

##### General Procedure

Iron powder (0.4 eq.) was added to a solution of *o*-nitrobenzaldehyde (**11a**–**11g**, 1.0 eq.) in EtOH (2.0 mL) followed by HCl (0.05 eq.) [19]. The reaction mixture was refluxed for 40 min followed by cooling to room temperature. The corresponding aldehyde or ketone (1.2 eq.) was added to the reaction mixture followed by KOH (1.2 eq.). The reaction mixture was stirred at RT for 30 min and refluxed for an additional 3 h. The reaction mixture was filtered, the filtrate was neutralized (HCl), extracted with EtOAc, and the combined extracts were dried and concentrated to dryness. The residue was subjected to FCC (EtOAc–hexane, 3:7) to afford substituted quinolines **5a**–**7g**.

*2-Phenylquinoline* (**5a**): Compound **5a** was prepared from *o*-nitrobenzaldehyde **11a** (151 mg, 1.00 mmol) according to the above procedure in 83% yield (171 mg, 0.83 mmol), obtained as a white powder, m.p. 78–79 °C. Satisfactory spectroscopic data identical to those previously reported [24].

*5-Chloro-2-phenylquinoline* (**5b**): Compound **5b** was prepared from *o*-nitrobenzaldehyde **11b** (200 mg, 1.07 mmol) according to the above procedure in 84% yield (215 mg, 0.90 mmol), obtained as a white powder, m.p. 90–91 °C. Although referenced in the SciFinder database as previously prepared (accessed on 17 April 2017), structure **5b** appears to be a new compound, since it was not published in the cited reference [25].

^1^H-NMR (500 MHz, CDCl_3_): δ 8.63 (d, *J* = 9 Hz, 1H), 8.19 (m, 2H), 8.11 (d, *J* = 8 Hz, 1H), 7.99 (d, *J* = 9 Hz, 1H), 7.71–7.59 (m, 2H), 7.56 (m, 2H), 7.50 (dd, *J* = 7, 7 Hz, 1H). ^13^C-NMR (125 MHz, CDCl_3_): δ 158.1, 149.2, 139.2, 133.8, 131.4, 129.9, 129.5, 129.2, 129.1, 127.8, 126.5, 125.5, 119.9. HPLC *t*_R_ = 24.4 ± 0.2 min (method B). UV (HPLC, CH_3_OH-H_2_O) λ_max_ (nm): 208, 262. FTIR (KBr) ν_max_ cm^−1^: 3058, 1611, 1593, 1580, 1547, 1486, 1461, 1396, 1316, 1278, 1201, 1025, 960, 814, 775, 692, 671. HRMS-EI *m*/*z* (%): calc. for C_15_H_10_NCl: 239.0502, measured 239.0507 (100), 204.08 (33).

*6-Chloro-2-phenylquinoline* (**5c**): Compound **5c** was prepared from *o*-nitrobenzaldehyde **11c** (200 mg, 1.07 mmol) according to the above procedure in 63% yield (161 mg, 0.670 mmol), obtained as a white powder, m.p. 109–110 °C. Satisfactory spectroscopic data identical to those previously reported [26].

*6-Bromo-2-phenylquinoline* (**5d**): Compound **5d** was prepared from *o*-nitrobenzaldehyde **11d** (59 mg, 0.25 mmol) according to the above procedure in 64% yield (47 mg, 0.16 mmol), obtained as an off-white powder, m.p. 123–125 °C. Satisfactory spectroscopic data identical to those previously reported [27].

*6-Hydroxy-2-phenylquinoline* (**5e**): Compound **5e** was prepared from *o*-nitrobenzaldehyde **11e** (200 mg, 1.19 mmol) according to the above procedure in 42% yield (112 mg, 0.51 mmol), obtained as a brown powder, m.p. 220–223 °C. Satisfactory spectroscopic data identical to those previously reported [28].

*6-Methoxy-2-phenylquinoline* (**5f**): Compound **5f** was prepared from *o*-nitrobenzaldehyde **11f** (150 mg, 0.82 mmol) according to the above procedure in 60% yield (115 mg, 0.49 mmol), obtained as a white powder, m.p. 135–137 °C. Satisfactory spectroscopic data identical to those previously reported [24].

*3-Phenylquinoline* (**6a**): Compound **6a** was prepared from *o*-nitrobenzaldehyde **11a** (200 mg, 1.32 mmol) according to the above procedure in 63% yield (172 mg, 0.84 mmol), obtained as a light yellow powder, m.p. 45–46 °C. Satisfactory spectroscopic data identical to those previously reported [19].

*5-Chloro-3-phenylquinoline* (**6b**): Compound **6b** was prepared from *o*-nitrobenzaldehyde **11b** (200 mg, 1.07 mmol) according to the above procedure in 80% yield (207 mg, 0.87 mmol), obtained as an off-white powder, m.p. 115–116 °C. Compound **6b** was previously synthesized using a different procedure but no spectroscopic data was reported [29].

^1^H-NMR (500 MHz, CDCl_3_): δ 9.18 (d, *J* = 2 Hz, 1H), 8.22 (d, *J* = 2 Hz, 1H), 8.08 (d, *J* = 9 Hz, 1H), 7.88 (d, *J* = 2 Hz, 1H), 7.71 (m, 2H), 7.66 (dd, *J* = 9, 2 Hz, 1H), 7.55 (m, 2H), 7.47 (dd, *J* = 7.5, 7.5 Hz, 1H). ^13^C-NMR (125 MHz, CDCl_3_): δ 150.8, 148.1, 137.7, 135.0, 131.7, 130.2, 129.5, 129.2, 128.7, 128.6, 127.8, 127.3, 126.4. HPLC *t*_R_ = 22.9 ± 0.2 min (method B). UV (HPLC, CH_3_OH-H_2_O) λ_max_ (nm): 208, 238, 257. FTIR (KBr) ν_max_ cm^−1^: 3053, 1487, 1456, 1182, 977, 900, 810, 758, 742, 694. HRMS-EI *m/z* (%): calc. for C_15_H_10_NCl: 239.0502, measured 239.0507 (100), 204.08 (15).

*6-Chloro-3-phenylquinoline* (**6c**): Compound **6c** was prepared from *o*-nitrobenzaldehyde **11c** (200 mg, 1.07 mmol) according to the above procedure in 64% yield (163 mg, 0.68 mmol), obtained as a white powder, m.p. 105–107 °C. Satisfactory spectroscopic data identical to those previously reported [30].

*6-Bromo-2-phenylquinoline* (**6d**): Compound **6d** was prepared from *o*-nitrobenzaldehyde **11d** (200 mg, 0.869 mmol) according to the above procedure in 40% yield (100 mg, 0.35 mmol), obtained as a light yellow powder, m.p. 116–118 °C. Satisfactory spectroscopic data identical to those previously reported [31].

*6-Hydroxy-3-phenylquinoline* (**6e**): Compound **6e** was prepared from *o*-nitrobenzaldehyde **11e** (200 mg, 1.19 mmol) according to the above procedure in 39% yield (102 mg, 0.460 mmol), obtained as a light brown powder, m.p. 223–225 °C.

^1^H-NMR (500 MHz, DMSO-*d*_6_): δ 10.09 (s, 1H), 8.99 (d, *J* = 2 Hz, 1H), 8.41 (s, 1H), 7.89 (d, *J* = 9 Hz, 1H), 7.84 (d, *J* = 7.5 Hz, 2H), 7.53 (dd, *J* = 7.5, 7.5 Hz, 2H), 7.44 (dd, *J* = 7.5, 7.5 Hz, 1H), 7.31 (dd, *J* = 2, 9 Hz, 1H), 7.23 (d, *J* = 2 Hz, 1H). ^13^C-NMR (125 MHz, DMSO-*d*_6_): δ 155.9, 146.1, 142.2, 137.4, 132.8, 131.1, 130.1, 129.2, 128.0, 127.1, 122.0, 108.7. HPLC *t*_R_ = 14.0 ± 0.2 min (method B). UV (HPLC, CH3OH-H2O) λ_max_ (nm): 206, 255, 342. FTIR (KBr) ν_max_ cm^−1^: 2952, 1620, 1496, 1457, 1376, 1246, 1217, 1166, 950, 898, 763, 701. HRMS-EI *m*/*z* (%): calc. for C_15_H_11_NO: 221.0841, measured 221.0838 (100).

*6-Methoxy-3-phenylquinoline* (**6f**): Compound **6f** was prepared from *o*-nitrobenzaldehyde **11f** (178 mg, 0.98 mmol) according to the above procedure in 53% yield (123 mg, 0.52 mmol), obtained as a yellow powder, m.p. 127–129 °C. Satisfactory spectroscopic data identical to those previously reported [32].

*6-Phenylquinoline* (**7a**): Compound **7a** was prepared from *o*-nitrobenzaldehyde **11g** (20 mg, 0.09 mmol) according to the above procedure in 62% yield (11 mg, 0.053 mmol), obtained as a light brown powder, m.p. 105–106 °C. Satisfactory spectroscopic data identical to those previously reported [33].

*2-Methyl-6-phenylquinoline* (**7b**): Compound **7b** was prepared from *o*-nitrobenzaldehyde **11g** (50 mg, 0.25 mmol) according to the above procedure in 52% yield (25 mg, 0.11 mmol), obtained as a yellow powder, m.p. 89–91 °C. Satisfactory spectroscopic data identical to those previously reported [34].

*3-Methyl-6-phenylquinoline* (**7c**): Compound **7c** was prepared from *o*-nitrobenzaldehyde **11g** (20 mg, 0.090 mmol) and propanal (50 µL) according to the above procedure in 52% yield (10 mg, 0.050 mmol), obtained as a light yellow oil.

^1^H-NMR (500 MHz, CDCl_3_): δ 8.78 (d, *J* = 1.5 Hz, 1H), 8.14 (d, *J* = 8.5 Hz, 1H), 7.97 (s, 1H), 7.93 (s, 1H), 7.91 (d, *J* = 2 Hz, 1H), 7.73–7.71 (m, 2H), 7.51 (dd, *J* = 7.5, 8.0 Hz, 1H), 7.41 (dd, *J* = 8.5, 7.5 Hz, 1H), 2.55 (s, 3H). ^13^C-NMR (125 MHz, CDCl_3_): δ 152.6, 146.1, 140.7, 139.5, 135.1, 131.1, 129.8, 129.1, 128.5, 128.4, 127.9, 127.7, 125.1, 19.0. HPLC *t*_R_ = 23.3 ± 0.2 min (method A). UV (HPLC, CH_3_CN-H_2_O) λ_max_ (nm): 210, 250. FTIR (KBr) ν_max_ cm^−1^: 2974, 1600, 1486, 1345, 1174, 900, 835, 759, 697, 570, 479. HRMS-FD *m*/*z* (%): calc. for C_16_H_13_N: 219.1048, measured 219.1051 (100).

*3-Ethyl-6-phenylquinoline* (**7d**): Compound **7d** was prepared from *o*-nitrobenzaldehyde **11g** (60 mg, 0.27 mmol) according to the above procedure in 82% yield (50 mg, 0.21 mmol), obtained as a yellow oil.

^1^H-NMR (500 MHz, CDCl_3_): δ 8.80 (s, 1H), 8.15 (d, *J* = 8.5 Hz, 1H), 7.98 (d, *J* = 9.5 Hz, 2H), 7.93 (d, *J* = 8.5 Hz, 1H), 7.73 (d, *J* = 7.5 Hz, 2H), 7.51 (dd, *J* = 7, 7.5 Hz, 2H), 7.41 (dd, *J* = 7.5, 7 Hz, 1H), 2.88 (q, *J* = 8, 7.5, 7.5 Hz, 2H), 1.39 (t, *J* = 7.5, 7.5 Hz, 3H).^13^C-NMR (125 MHz, CDCl_3_): δ 151.9, 146.2, 140.7, 139.6, 137.3, 134.0, 129.6, 129.2, 128.6, 127.9, 127.7, 125.3, 26.5, 15.4. HPLC *t*_R_ = 27.2 ± 0.2 min (method A). UV (HPLC, CH_3_CN-H_2_O) λ_max_ (nm): 210, 252. FTIR (KBr) ν_max_ cm^−1^: 2965, 1599, 1487, 1348, 1179, 908, 837, 757, 697, 627, 570. HRMS-FD *m*/*z* (%): calc. for C_17_H_15_N: 233.1205, measured 233.1205 (100).

*3-Isopropyl-6-phenyl-quinoline* (**7e**): Compound **7e** was prepared from *o*-nitrobenzaldehyde **11g** (60 mg, 0.27 mmol according to the above procedure in 69% yield (45 mg, 0.18 mmol), obtained as a yellow oil.

^1^H-NMR (500 MHz, CDCl_3_): δ 8.85 (s, 1H), 8.17 (d, *J* = 8.5 Hz, 1H), 7.99 (d, *J* = 9.5 Hz, 2H), 7.93 (d, *J* = 10 Hz, 1H), 7.74–7.72 (m, 1H), 7.51 (dd, *J* = 7.5, 8 Hz, 2H), 7.41 (dd, *J* = 7.5, 7.5 Hz, 1H), 3.19–3.13 (m, 1H), 1.41 (s, 3H), 1.40 (s, 3H).^13^C-NMR (125 MHz, CDCl_3_): δ 151.3, 146.4, 141.7, 140.7, 139.4, 132.3, 129.6, 129.1, 128.6, 128.5, 127.8, 127.6, 125.4, 32.1, 23.8. HPLC *t*_R_ = 30.1 ± 0.2 min (method A). UV (HPLC, CH_3_CN-H_2_O) λ_max_ (nm): 210, 252. FTIR (KBr) ν_max_ cm^−1^: 2960, 1599, 1486, 1351, 1178, 1077, 975, 909, 837, 757, 697, 473. HRMS-FD *m*/*z* (%): calc. for C_18_H_17_N: 247.1361, measured 247.1372 (100).

*3,6-Diphenylquinoline* (**7f**): Compound **7f** was prepared from *o*-nitrobenzaldehyde **11g** (40 mg, 0.18 mmol) according to the above procedure in 100% yield (50 mg, 0.18 mmol), obtained as a light yellow powder, m.p. 131–133 °C.

^1^H-NMR (500 MHz, CDCl_3_): δ 9.20 (s, 1H), 8.34 (s, 1H), 8.23 (d, *J* = 9 Hz, 1H), 8.10 (s, 1H), 8.00 (d, *J* = 9 Hz, 1H), 7.76–7.74 (m, 4H), 7.57–7.51 (m, 4H), 7.48–7.42 (m, 2H).^13^C-NMR (125 MHz, CDCl_3_): δ 150.0, 146.9, 140.4, 139.9, 138.0, 134.4, 133.6, 129.8, 129.4, 129.3, 129.2, 128.4, 128.3, 128.0, 127.6, 127.5, 125.8. HPLC *t*_R_ = 30.1 ± 0.2 min (method A). UV (HPLC, CH_3_CN-H_2_O) λ_max_ (nm): 210, 280. FTIR (KBr) ν_max_ cm^−1^: 3052, 1487, 1348, 1077, 910, 834, 758, 699, 571, 531, 497, 470. HRMS-FD *m*/*z* (%): calc. for C_21_H_15_N: 281.1205, measured 281.1218 (100).

*2,6-Diphenylquinoline* (**7g**): Compound **7g** was prepared from *o*-nitrobenzaldehyde **11g** (20 mg, 0.090 mmol) according to the above procedure in 62% yield (15 mg, 0.053 mmol), obtained as a light brown powder, m.p. 198–200 °C. Satisfactory spectroscopic data identical to those previously reported [35].

#### 3.2.2. Synthesis Isoquinolines **9b**, **9c** and **10b**

##### Isoquinolines ***9b*** and ***9c***

Aminoacetaldehyde dimethylacetal (3.0 eq.) was added to a solution of bromobenzaldehyde **13b** or **13c** (1.0 eq.) in toluene (30 mL). Each reaction mixture was refluxed (Dean–Stark apparatus) at 120 °C. After consumption of the starting material, each reaction mixture was concentrated to dryness, then dissolved in conc. H_2_SO_4_ (2 mL) and added to a cold solution of P_2_O_5_ in conc. H_2_SO_4_ (0.5 mL). Each reaction mixture was heated at 160 °C for 30 min, allowed to cool to RT, neutralized with NaOH (10 M), extracted with EtOAc, and concentrated to dryness. Each residue was subjected to FCC to afford 6-bromoisoquinoline (**14b**, 30 mg, 0.14 mmol, 14%) and 7-bromoisoquinoline (**14c**, 99 mg, 0.47 mmol, 22%) [20,21]. Ethylchloroformate (1.0 eq.) was added to a solution of isoquinoline **14b** or **14c** (1.0 eq.) in DCM at 0 °C and stirred at the same temperature for 30 min, followed by addition of 2-trimethylsilylthiazole (1.0 eq.). Each reaction mixture was stirred at RT for 3 h, concentrated to dryness, and each residue was subjected to FCC. Each product was dissolved in benzene (5 mL), *o*-chloranil (1.0 eq.) was added, and each reaction mixture was refluxed for 5 h. Each reaction mixture was diluted with 5% NaOH (10 mL), extracted with DCM, and concentrated to dryness. Each reaction mixture residue was subjected to FCC to afford the products **9b** and **9c**.

*6-Bromo-1-(2-thiazolyl)isoquinoline* (**9b**): 6-Bromoisoquinoline (**14b**, 30 mg, 0.14 mmol) was synthesized starting from 4-bromobenzaldehyde (**13c**, 200 mg, 1.08 mmol) in 14% yield. Compound **9b** was synthesized starting from 6-bromoisoquinoline (**14b**, 100 mg, 0.48 mmol) in 15% yield over two steps (21 mg, 0.07 mmol), obtained as an orange powder, m.p. 103–105 °C.

^1^H-NMR (500 MHz, CDCl_3_): δ 9.77 (d, *J* = 9 Hz, 1H), 8.58 (d, *J* = 5.5 Hz, 1H), 8.06 (m, 2H), 7.79 (dd, *J* = 2, 9 Hz, 1H), 7.61 (d, *J* = 5.5 Hz, 1H), 7.53 (d, *J* = 3 Hz, 1H). ^13^C-NMR (125 MHz, CDCl_3_): δ 170.6, 149.9, 144.4, 143.0, 138.7, 132.3, 130.1, 129.1, 125.7, 124.0, 122.5, 121.2. HPLC *t*_R_ = 22.4 ± 0.2 min (method B). UV (HPLC, CH_3_OH-H_2_O) λ_max_ (nm): 240, 345. FTIR (KBr) ν_max_ cm^−1^: 2924, 2852, 1782, 1608, 1546, 1426, 1246, 1183, 947, 880, 810. HRMS-EI *m/z* (%): calc. for C_12_H_7_N_2_SBr: 291.9493, measured 291.9494 (100), 289.95 (95), 247.88 (53), 211.03 (21), 99.96 (39).

*7-Bromo-1-(2-thiazolyl)isoquinoline* (**9c**): 7-Bromoisoquinoline (**14c**, 99 mg, 0.47 mmol) was synthesized starting from compound **13b** in 22% yield [20]. Compound **9c** was synthesized starting from 7-bromoisoquinoline **(14c**, 100 mg, 0.48 mmol) in 24% yield over two steps (35 mg, 0.12 mmol), obtained as an off-white powder m.p. 110–112 °C.

^1^H-NMR (500 MHz, CDCl_3_): δ 10.12 (s, 1H), 8.58 (d, *J* = 5.5 Hz, 1H), 8.08 (d, *J* = 3 Hz, 1H), 7.80 (dd, *J* = 9, 2 Hz, 1H), 7.73 (d, *J* = 9 Hz, 1H), 7.67 (d, *J* = 5.5 Hz, 1H), 7.53 (d, *J* = 3 Hz, 1H). ^13^C-NMR (500 MHz, CDCl_3_): δ 170.3, 148.6, 144.5, 142.3, 136.0, 134.2, 130.5, 128.6, 126.3, 123.1, 122.5, 122.1. HPLC *t*_R_ = 21.0 ± 0.2 min (method B). UV (HPLC, CH_3_OH-H_2_O) λ_max_ (nm): 240, 302, 350. FTIR (KBr) ν_max_ cm^−1^: 3089, 1781, 1573, 1544, 1491, 1425, 1247, 1103, 947, 850, 813, 732. HRMS-EI *m/z* (%): calc. for C_12_H_7_N_2_SBr: 291.9493, measured 291.9483 (100), 289.95 (99), 247.94 (34), 211.03 (32), 99.96 (24).

*1-Phenylisoquinoline* (**10a**): Iodine (18 mg, 0.073 mmol) was added to a solution of compound **15a** (12 mg, 0.073 mmol) in THF (2 mL) and stirred at RT. After 30 min, Fe powder (16 mg, 0.29 mmol) followed by PhMgCl (2 M in THF, 43 µL, 0.087 mmol) were added and stirred at RT. After 6 h, the reaction mixture was diluted with ice-cold water (20 mL) and extracted with EtOAc. The combined organic extracts were dried and concentrated to dryness to afford **10a** in 67% yield (10 mg, 0.050 mmol) as an off-white powder, m.p. 91–92 °C. Satisfactory spectroscopic data identical to those previously reported [36].

*7-Bromo-1-phenylisoquinoline* (**10b**): Iodine (18 mg, 0.073 mmol) was added to a solution of compound **15b** (40 mg, 0.16 mmol) in THF (2 mL) and stirred at RT for 30 min followed by addition of Fe powder (16 mg, 0.294 mmol) and PhMgCl (2 M in THF, 43 µL, 0.087 mmol). The reaction mixture was stirred at RT for 6 h followed by dilution with ice-cold water, extraction with EtOAc, and concentration to dryness to afford **10b** in 47% yield (21 mg, 0.07 mmol) as yellow gum [36].

^1^H-NMR (500 MHz, CDCl_3_): δ 8.64 (d, *J* = 6 Hz, 1H), 8.07 (d, *J* = 2 Hz, 1H), 7.99 (d, *J* = 9 Hz, 1H), 7.68 (dd, *J* = 2, 7.5 Hz, 2H), 7.62 (dd, *J* = 2, 9 Hz, 1H), 7.58–7.53 (m, 4H). ^13^C-NMR (500 MHz, CDCl_3_): δ 161.2, 143.6, 139.3, 138.2, 130.9, 130.1, 129.7, 129.3, 129.1, 128.7, 125.3, 125.1, 119.0. HPLC *t*_R_ = 23.4 ± 0.2 min (method B). UV (HPLC, CH_3_OH-H_2_O) λ_max_ (nm): 220, 258, 328. FTIR (KBr) ν_max_ cm^−1^: 3061, 3033, 2923, 1480, 1429, 1261, 1091, 1042, 1008, 903, 800, 728, 697. HRMS-EI *m*/*z* (%): calc. for C_15_H_10_NBr: 284.9976, measured 284.9969 (10), 237.08 (26), 184.04 (41), 91.05 (100), 82.07 (86).

### 3.3. Antifungal Activity

The antifungal activity of brassinin (**1**), camalexin (**3a**), quinolines **5a**–**8**, and isoquinolines **9a**–**10b** against *L. maculans* was evaluated using a mycelial radial growth assay carried out in PDA [23]. Cultures of *L. maculans* were grown on potato dextrose agar (PDA) plates (10 cm diameter) at 23 ± 1 °C under constant light (fluorescent 32W T8 48" daylight) for seven days. DMSO solutions of each compound (0.50, 0.20 and 0.10 mM) in PDA (1% DMSO concentration in PDA) were added to each well (six-well plates, 1.5 mL per well) and inoculated with mycelia (4 mm diameter plugs cut from the edges of 7-day-old solid cultures, placed upside down on the center of each well). Control cultures containing 1% DMSO in PDA were prepared similarly. Plates were incubated under constant light and the diameter of mycelial mat (mm) in each well was measured after five days of incubation. Each assay was conducted in triplicate and repeated twice. The percentage of growth inhibition was calculated as reported in Table 2.

### 3.4. Fungal Cultures, Preparation of Cell-Free Protein Extracts, and BOLm Activity

Liquid cultures of *L. maculans* (isolate UAHM 9410) were handled as described previously. In brief, fungal spores were subcultured on V8 agar under continuous light at 23 ± 1 °C; after 15 days, fungal spores were collected aseptically and stored at −20 °C. Liquid cultures were initiated by inoculating minimal media (100 mL) with fungal spores at 10^7^/mL in Erlenmeyer flasks, followed by incubation on a shaker under constant light at 23 °C for 48 h; 3-phenylindole (0.050 mM in DMSO) was added to each flask to induce BOLm, and cultures were incubated for an additional 24 h. Cultures were gravity filtered to separate mycelia from culture broth, the mycelia was washed with distilled water, wrung, and stored at −20 °C.

Frozen mycelia (1.2 g) from *L. maculans* (obtained as reported above) were ground in ice-cold extraction buffer (5 mL, mortar and pestle) for 5 min at 4 °C. The extraction buffer consisted of diethanolamine (DEA, 25 mM, pH ~ 8.3), 10% (*v*/*v*) glycerol, d,l-dithiothreitol (DTT, 1 mM), and 1/200 (*v*/*v*) protease inhibitor cocktail (P-8215, Sigma-Aldrich Canada). The homogenate was centrifuged at 4 °C for 30 min at 50,000 *g*. The resulting supernatant was dialyzed three times (twice with 300 mL of dialyzing buffer for 3 h each time and then using 400 mL buffer for 12 h, using dialyzing cassettes in buffer pH 8.3, diethanolamine, 25 mM, 5% glycerol, *v*/*v*, 10% triton X-100 in deionized water). Dialyzed cell-free protein extracts were used for determination of the specific activity of BOLm and for testing inhibitory activity of compounds.

Protein concentrations were determined as described by Bradford using the Coomassie Brilliant Blue method with BSA as a standard (optical density (OD) was measured at 595 nm).

#### Determination of BOLm Activity

The reaction mixture contained diethanolamine (20 mM, pH ~8.3), DTT (0.10 mM), 0.1% (*v*/*v*) triton X-100, brassinin (**1**, 0.10 mM), PMS (0.10 mM), and cell-free protein extract (50.0 µL) in a total volume of 1.0 mL. The reaction was carried out at 24 °C for 20 min. The product was extracted with EtOAc (4 mL) and the extract was concentrated to dryness; the residue was dissolved in CH_3_CN (200 µL) and analyzed by HPLC-DAD. The amount of indole-3-carboxaldehyde (**2**) in the reaction assay was determined using a calibration curve built with pure indole-3-carboxaldehyde (λ_max_ 220 nm).

## 4. Conclusions

Overall, our results indicate that at higher concentration (0.30 mM) 3-ethyl-6-phenylquinoline (**7d**) inhibited BOLm activity to a larger extent than camalexin (**3a**) and displayed lower antifungal activity against *L. maculans*, both very desirable characteristics. Furthermore, no direct correlation was found between BOLm inhibitory activity of quinolines and their antifungal activity against *L. maculans*. Nevertheless, considering the antifungal activity against *L. maculans,* compounds with a diversity of scaffolds need to be synthesized and assayed before the desirable paldoxin activity of different scaffolds can be predicted. To this end, availability of the crystal structure of BOLm would be of great assistance.

The design of new compounds with new mechanisms of action to protect crops against fungi is expensive and time consuming, providing rather unpredictable outcomes. Nonetheless, the discovery of novel agrochemicals useful for sustainable crop treatments is crucial. It is expected that in the future, broad range fungicides will be replaced with selective crop treatments and that paldoxins will be a rational approach to treat specific fungal diseases such as that caused by *L. maculans*. Further work to uncover paldoxins that can replace current fungicides and prevent crop infestations with *L. maculans* is necessary.

## Figures and Tables

**Table 1:**
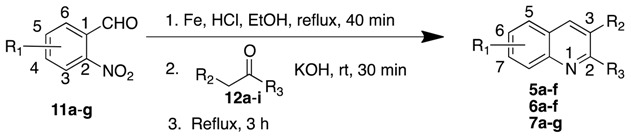
Synthesis of substituted quinolines **5a**–**5f**, **6a**–**6f** and **7a**–**7g**.

Starting Material	R_1_ (C#)	12	R_2_	R_3_	Product	%Yield (Reported Yield)
**11a**	H	**12a**	H	Ph	**5a** ^a^	83 (99)
**11b**	6-Cl	**12a**	H	Ph	**5b** ^b^	84
**11c**	5-Cl	**12a**	H	Ph	**5c** ^b^	63
**11d**	5-Br	**12a**	H	Ph	**5d** ^b^	64
**11e**	5-OH	**12a**	H	Ph	**5e** ^b^	42
**11f**	5-OMe	**12a**	H	Ph	**5f** ^b^	60
**11a**	H	**12b**	Ph	H	**6a** ^a^	63 (87)
**11b**	6-Cl	**12b**	Ph	H	**6b** ^b^	80
**11c**	5-Cl	**12b**	Ph	H	**6c** ^b^	64
**11d**	5-Br	**12b**	Ph	H	**6d** ^b^	36
**11e**	5-OH	**12b**	Ph	H	**6e** ^c^	39
**11f**	5-OMe	**12b**	Ph	H	**6f** ^b^	53
**11g**	5-Ph	**12c**	H	H	**7a** ^b^	62
**11g**	5-Ph	**12d**	Me	H	**7b** ^b^	52
**11g**	5-Ph	**12e**	H	Me	**7c** ^c^	52
**11g**	5-Ph	**12f**	Et	H	**7d** ^c^	82
**11g**	5-Ph	**12g**	*i*-Pro	H	**7e** ^c^	69
**11g**	5-Ph	**12h**	Ph	H	**7f** ^b^	quantitative
**11g**	5-Ph	**12i**	H	Ph	**7g** ^c^	62

^a^ Previously synthesized using this method [19]; ^b^ Previously synthesized by different methods; ^c^ New compounds.

**Table 2:** Antifungal activity ^a^ of the phytoalexins brassinin (**1**), camalexin (**3a**), quinolines **5a**–**8**, and isoquinolines **9a**–**10b** against *Leptosphaeria maculans*.

Compound (#)	Inhibition ± SD (%) ^a^		
	0.50 mM	0.20 mM	0.10 mM
Brassinin (**1**)	50 ± 3 ^i,g^	27 ± 0 ^l^	14 ± 3 ^j^
Camalexin (**3a**)	100 ± 0 ^c^	41 ± 2 ^i,j^	24 ± 3 ^i^
2-Phenylquinoline (**5a**)	54 ± 2 ^h^	31 ± 4 ^k,l^	16 ± 5 ^j^
5-Chloro-2-phenylquinoline (**5b**)	39 ± 2 ^l^	18 ± 4 ^m,n^	9 ± 2 ^k^
6-Chloro-2-phenylquinoline (**5c**)	26 ± 4 ^m^	18 ± 5 ^m,n^	5 ± 2 ^l^
6-Bromo-2-phenylquinoline (**5d**)	27 ± 4 ^m^	14 ± 4 ^n,^^o^	0 ^m^
6-Hydroxy-2-phenylquinoline (**5e**)	68 ± 5 ^e,f^	42 ± 4 ^h,i,j^	32 ± 4 ^f,g,h^
6-Methoxy-2-phenylquinoline (**5f**)	36 ± 4 ^l^	20 ± 4 ^m^	8 ± 2 ^k^
3-Phenylquinoline (**6a**)	100 ± 0 ^c^	77 ± 4 ^d^	39 ± 7 ^f^
5-Chloro-3-phenylquinoline (**6b**)	17 ± 3 ^n^	11 ± 2 ^o^	8 ± 4 ^k,l^
6-Chloro-3-phenylquinoline (**6c**)	28 ± 3 ^m^	17 ± 3 ^m,n^	5 ± 2 ^l^
6-Bromo-3-phenylquinoline (**6d**)	45 ± 2 ^k^	34 ± 2 ^k^	25 ± 0 ^i^
6-Hydroxy-3-phenylquinoline (**6e**)	73 ± 5 ^e^	45 ± 4 ^h,i^	30 ± 4 ^g,h^
6-Methoxy-3-phenylquinoline (**6f**)	100 ± 0 ^c^	68 ± 5 ^e^	55 ± 5 ^c^
6-Methyl-3-phenylquinoline (**6g**)	69 ± 3 ^e,f^	61 ± 2 ^f^	47 ± 0 ^e^
6-Phenylquinoline (**7a**)	64 ± 0 ^g^	55 ± 0 ^g^	30 ± 0 ^h^
2-Methyl-6-phenylquinoline (**7b**)	68 ± 2 ^f^	62 ± 3 ^f^	48 ± 3 ^d,e^
3-Methyl-6-phenylquinoline (**7c**)	74 ± 2 ^e^	67 ± 4 ^e^	53 ± 0 ^c^
3-Ethyl-6-phenylquinoline (**7d**)	67 ± 3 ^f^	61 ± 3 ^f^	48 ± 3 ^d,e^
3-Isopropyl-6-phenylquinoline (**7e**)	72 ± 3 ^e^	66 ± 3 ^e^	56 ± 3 ^c^
2,6-Diphenylquinoline (**7f**)	49 ± 4 ^i,j,k^	40 ± 0 ^j^	33 ± 0 ^f,g^
3,6-Diphenylquinoline (**7g**)	0 ^o^	0 ^p^	0 ^m^
2-(2-Thiazolyl)quinoline (**8**)	54 ± 3 ^h^	46 ± 4 ^h^	8 ± 3 ^k,l^
1-(2-Thiazolyl)isoquinoline (**9a**)	100 ± 0 ^c^	100 ± 0 ^c^	52 ± 4 ^c,d^
6-Bromo-1-(2-thiazolyl)isoquinoline (**9b**)	75 ± 6 ^d,e^	54 ± 3 ^g^	15 ± 4 ^j^
7-Bromo-1-(2-thiazolyl)isoquinoline (**9c**)	82 ± 5 ^d^	63 ± 3 ^e,f^	25 ± 6 ^i^
1-Phenylisoquinoline (**10a**)	54 ± 5 ^h,i^	32 ± 4 ^k^	30 ± 3 ^h^
7-Bromo-1-phenylisoquinoline (**10b**)	47 ± 3 ^j,k^	31 ± 4 ^k,l^	12 ± 2 ^j^

^a^ The percentage of inhibition was calculated using the formula: % inhibition = 100 − [(growth on amended/growth in control) × 100]; values are averages of six independent experiments conducted in triplicate; ^b^ for statistical analysis, one-way ANOVA tests were performed followed by Tukey’s test with adjusted α set at 0.05; *n* = 6; different letters in the same column (c–p) indicate significant differences (*p* < 0.05).

**Table 3:** Inhibitory effect of camalexin (**3a**) and quinolines **5a**–**8** and isoquinolines **9a**–**10b** on BOLm activity.

Potential Inhibitor (#) ^a^	% Inhibition ^b^		Potential Inhibitor (#) ^a^	% Inhibition ^b^	
	0.10 mM	0.30 mM		0.10 mM	0.30 mM
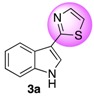			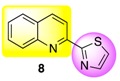		
	30 ± 4 ^c^	53 ± 4 ^d^		n.i.	n.i.
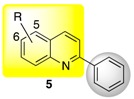			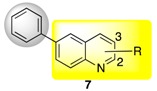		
**5a** R = H	n.i.	n.i.	**7a** R = H	16 ± 2 ^d,e^	36 ± 2 ^e,f^
**5b** R = 5-Cl	n.i.	n.i.	**7b** R = 2-Me	n.i.	n.i.
**5c** R = 6-Cl	n.i.	n.i.	**7c** R = 3-Me	19 ± 4 ^d^	33 ± 6 ^e,f^
**5d** R = 6-Br	n.i.	n.i.	**7d** R = 3-Et	30 ± 4 ^c^	64 ± 5 ^c^
**5e** R = 6-OH	11 ± 3 ^e^	22 ± 1 ^h^	**7e** R = 3-CH(CH_3_)_2_	11 ± 3 ^e^	33 ± 5 ^e,f^
**5f** R = 6-OMe	n.i.	n.i.	**7f** R = 2-Ph	10 ± 2 ^e^	19 ± 1 ^h^
			**7g** R = 3-Ph	12 ± 1 ^e^	25 ± 1 ^g,h^
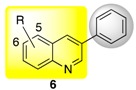			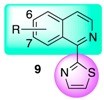		
**6a** R = H	14 ± 2 ^d,e^	30 ± 4 ^f,g^	**9a** R = H	n.i.	n.i.
**6b** R = 5-Cl	n.i.	n.i.	**9b** R = 6-Br	n.i.	n.i.
**6c** R = 6-Cl	n.i.	n.i.	**9c** R = 7-Br	n.i.	n.i.
**6d** R = 6-Br	n.i.	n.i.	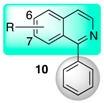		
**6e** R = 6-OH	20 ± 4 ^d^	34 ± 2 ^e,f^			
**6f** R = 6-OMe	19 ± 1 ^d^	40 ± 3 ^e^			
**6g** R = 6-Me	10 ± 3 ^e^	32 ± 3 ^f^			
			**10a** R = H	n.i.	n.i.
			**10b** R = 7-Br	n.i.	n.i.

^a^ Brassinin (**1**) was used as substrate (0.10 mM) and PMS (0.10 mM) as cofactor in all experiments. BOLm activity was measured under standard conditions (described in Section 3.4) in the presence of potential inhibitors (0.10 and 0.30 mM). The specific activity of BOLm (24 ± 1 nmol/mg/min) was comparable in all assays; ^b^ Inhibition is expressed as a percentage of the BOLm activity (100%) of cell-free protein extracts containing brassinin (**1**) and inhibitor; results are means and standard deviations of experiments conducted in triplicate; n.i. = no inhibition. For statistical analysis, one-way ANOVA tests were performed followed by Tukey’s test; *n* = 6; different superscript letters within the same column (c–h) indicate significant differences (*p* < 0.05).

## Supplementary Materials

The following are available online. ^1^H- and ^13^C-NMR spectra of all new compounds: **5b**, **6b**, **6e**, **7c**, **7d**, **7e**, **7g**, **9b**, **9c** and **10b**.

## References

[1] Pedras M.S.C., Minic Z., Jha M. (2008). Brassinin oxidase, a fungal detoxifying enzyme to overcome a plant defense—Purification, characterization and inhibition. FEBS J..

[2] Pedras M.S.C., Minic Z., Sarma-Mamillapalle V.K. (2011). Brassinin oxidase mediated transformation of the phytoalexin brassinin: Structure of the elusive co-product, deuterium isotope effect and stereoselectivity. Bioorg. Med. Chem..

[3] Pedras M.S.C., Yaya E.E., Glawischnig E. (2011). The phytoalexins from cultivated and wild crucifers: Chemistry and biology. Nat. Prod. Rep..

[4] Pedras M.S.C. (2005). Protecting plants against fungal diseases. Can. Chem. News.

[5] Pedras M.S.C., Minic Z., Sarma-Mamillapalle V.K., Suchy M. (2010). Discovery of inhibitors of brassinin oxidase based on the scaffolds of the phytoalexins brassilexin and wasalexin. Bioorg. Med. Chem..

[6] Pedras M.S.C., Minic Z., Sarma-Mamillapalle V.K. (2009). Synthetic inhibitors of the fungal detoxifying enzyme brassinin oxidase based on the phytoalexin camalexin scaffold. J. Agric. Food Chem..

[7] Pedras M.S.C., Abdoli A. (2017). Pathogen inactivation of cruciferous phytoalexins: Detoxification reactions, enzymes and inhibitors. RSC Adv..

[8] Pedras M.S.C., Jha M. (2006). Toward the control of *Leptosphaeria maculans*: Design, syntheses, biological activity, and metabolism of potential detoxification inhibitors of the crucifer phytoalexin brassinin. Bioorg. Med. Chem..

[9] Commercial Agriculture Fungicides are Classified According to Their Target Sites by the International Fungicide Resistance Action Committee (FRAC), cf. http://www.frac.info/docs/default-source/publications/frac-code-list/frac-code-list-2016.pdf?sfvrsn=2.

[10] Lamberth C., Walter H., Kessabi F.M., Quaranta L., Beaudegnies R., Trah S., Jeanguenat A., Cederbaum F. (2015). The significance of organosulfur compounds in crop protection: Current examples from fungicide research. Phosphorus Sulfur.

[11] Lamberth C., Kessabi F.M., Beaudegnies R., Quaranta L., Trah S., Berthon G., Cederbaum F., Knauf-Beiter G., Grasso V., Bieri S. (2014). Synthesis and fungicidal activity of quinolin-6-yloxyacetamides, a novel class of tubulin polymerization inhibitors. Bioorg. Med. Chem..

[12] Ayer W.A., Peter A.C., Ma Y., Miao S. (1992). Synthesis of camalexin and related phytoalexins. Tetrahedron.

[13] Dondoni A., Dall’Occo T., Galliani G., Mastellari A., Medici A. (1984). Addition of 2-silylazoles to heteroaryl cations. Synthesis of unsymetrical azadiaryls. Tetrahedron Lett..

[14] Batista V.F., Pinto D.C.G.A., Silva A.M.S. (2016). Synthesis of quinolines: A green perspective. ACS Sustain. Chem. Eng..

[15] Chelucci G., Porcheddu A. (2017). Synthesis of quinolines via a metal-catalyzed dehydrogenative *N*-heterocyclization. Chem. Rec..

[16] Prajapati S.M., Patel K.D., Vekariya R.H., Panchal S.N., Patel H.D. (2014). Recent advances in the synthesis of quinolines: A review. RSC Adv..

[17] Cieslik W., Serda M., Kurczyk A., Musiol R. (2013). Microwave assisted synthesis of monoazanaphthalene scaffolds. Curr. Org. Chem..

[18] Li W.Y., Xiong X.Q., Zhao D.M., Shi Y.F., Yang Z.H., Yu C., Fan P.W., Cheng M.S., Shen J.K. (2012). Quinoline-3-carboxamide derivatives as potential cholesteryl ester transfer protein inhibitors. Molecules.

[19] Li A.H., Ahmed E., Chen X., Cox M., Crew A.P., Dong H.Q., Jin M., Ma L., Panicker B., Siu K.W. (2007). A highly effective one-pot synthesis of quinolines from *o*-nitroarylcarbaldehydes. Org. Biomol. Chem..

[20] Jiang R., Duckett D., Chen W., Habel J., Ling Y.Y., LoGrasso P., Kamenecka T.M. (2007). 3,5-Disubstituted quinolines as novel c-Jun N-terminal kinase inhibitors. Bioorg. Med. Chem. Lett..

[21] Czakó B., Kürti L., Mammoto A., Ingber D.E., Corey E.J. (2009). Discovery of potent and practical antiangiogenic agents inspired by cortistatin A. J. Am. Chem. Soc..

[22] Korn T.J., Schade M.A., Cheemala M.N., Wirth S., Guevara S.A., Cahiez G., Knochel P. (2006). Cobalt-catalyzed cross-coupling reactions of heterocyclic chlorides with arylmagnesium halides and of polyfunctionalized arylcopper reagents with aryl bromides, chlorides, fluorides and tosylates. Synthesis.

[23] Pedras M.S.C., Abdoli A. (2013). Metabolism of the phytoalexins camalexins, their bioisosteres and analogues in the plant pathogenic fungus *Alternaria brassicicola*. Bioorg. Med. Chem..

[24] Movassaghi M., Hill M.D. (2006). Synthesis of substituted pyridine derivatives via the ruthenium-catalyzed cycloisomerization of 3-azadienynes. J. Am. Chem. Soc..

[25] Ji X., Huang H., Li Y., Chen H., Jiang H. (2012). Palladium-catalyzed sequential formation of C-C bonds: Efficient assembly of 2-substituted and 2,3-disubstituted quinolines. Angew. Chem. Int. Ed. Engl..

[26] Shi D., Rong L., Shi C., Zhuang Q., Wang X., Tu S., Hu H. (2005). Low-valent titanium reagent-promoted intramolecular reductive coupling reactions of ketomalononitriles: A facile synthesis of benzo[4,5]indene, acridine and quinoline derivatives. Synthesis.

[27] Huo Z., Gridnev I.D., Yamamoto Y. (2010). A method for the synthesis of substituted quinolines via electrophilic cyclization of 1-azido-2-(2-propynyl)benzene. J. Org. Chem..

[28] Meléndez Gómez C.M., Kouznetsov V.V., Sortino M.A., Álvarez S.L., Zacchino S.A. (2008). In vitro antifungal activity of polyfunctionalized 2-(hetero)arylquinolines prepared through imino Diels-Alder reactions. Bioorg. Med. Chem..

[29] Colomb J., Billard T. (2013). Palladium-catalyzed desulfitative arylation of 3-haloquinolines with arylsulfinates. Tetrahedron Lett..

[30] Wang Y., Xin X., Liang Y., Lin Y., Zhang R., Dong D. (2009). A facile and efficient one-pot synthesis of substituted quinolines from α-arylamino ketones under vilsmeier conditions. Eur. J. Org. Chem..

[31] Saunthwal R.K., Patel M., Verma A.K. (2016). Regioselective synthesis of C-3-functionalized quinolines via Hetero-Diels-Alder cycloaddition of azadienes with terminal alkynes. J. Org. Chem..

[32] Monrad R.N., Madsen R. (2011). Ruthenium-catalysed synthesis of 2- and 3-substituted quinolines from anilines and 1,3-diols. Org. Biomol. Chem..

[33] Shrestha B., Thapa S., Gurung S.K., Pike R.A.S., Giri R. (2016). General copper-catalyzed coupling of alkyl-, aryl-, and alkynylaluminum reagents with organohalides. J. Org. Chem..

[34] Mongin F., Mojovic L., Guillamet B., Trécourt F., Quéguiner G. (2002). Cross-coupling reactions of phenylmagnesium halides with fluoroazines and fluorodiazines. J. Org. Chem..

[35] Li C., Li J., An Y., Peng J., Wu W., Jiang H. (2016). Palladium-catalyzed allylic C-H oxidative annulation for assembly of functionalized 2-substituted quinoline derivatives. J. Org. Chem..

[36] Larivée A., Mousseau J.J., Charette A.B. (2008). Palladium-catalyzed direct C–H arylation of *N*-iminopyridinium ylides: Application to the synthesis of (±)-anabasine. J. Am. Chem. Soc..

