# Supplementary material for: Inhibitors of the Detoxifying Enzyme of the Phytoalexin Brassinin Based on Quinoline and Isoquinoline Scaffolds
Source: Molecules. 2017 Aug 14;22(8):1345. doi: 10.3390/molecules22081345 (PMC6152025; doi:10.3390/molecules22081345)
Supplement: Supplementary file 1 [file molecules-22-01345-s001.pdf]

Inhibitors of the detoxifying enzyme of the phytoalexin brassinin based on quinoline and isoquinoline scaffolds

M. Soledade C. Pedras*, Abbas Abdoli and Vijay K. Sarma-Mamillapalle

Department of chemistry, University of Saskatchewan, 110 Science place, Saskatoon, SK, S7N5C9 Canada

s.pedras@usask.ca

^{1}H and ^{13}C NMR of new compounds 5b, 6b, 6e, 7c, 7d, 7e, 7g, 9b, 9c and 10b.

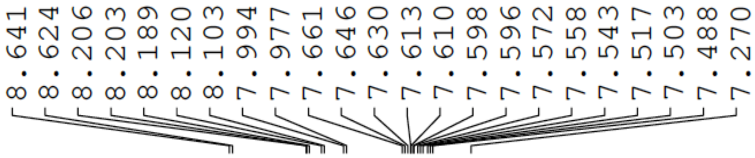

^{1}H NMR ($CDCl_3$) compound 5b

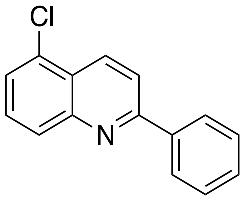

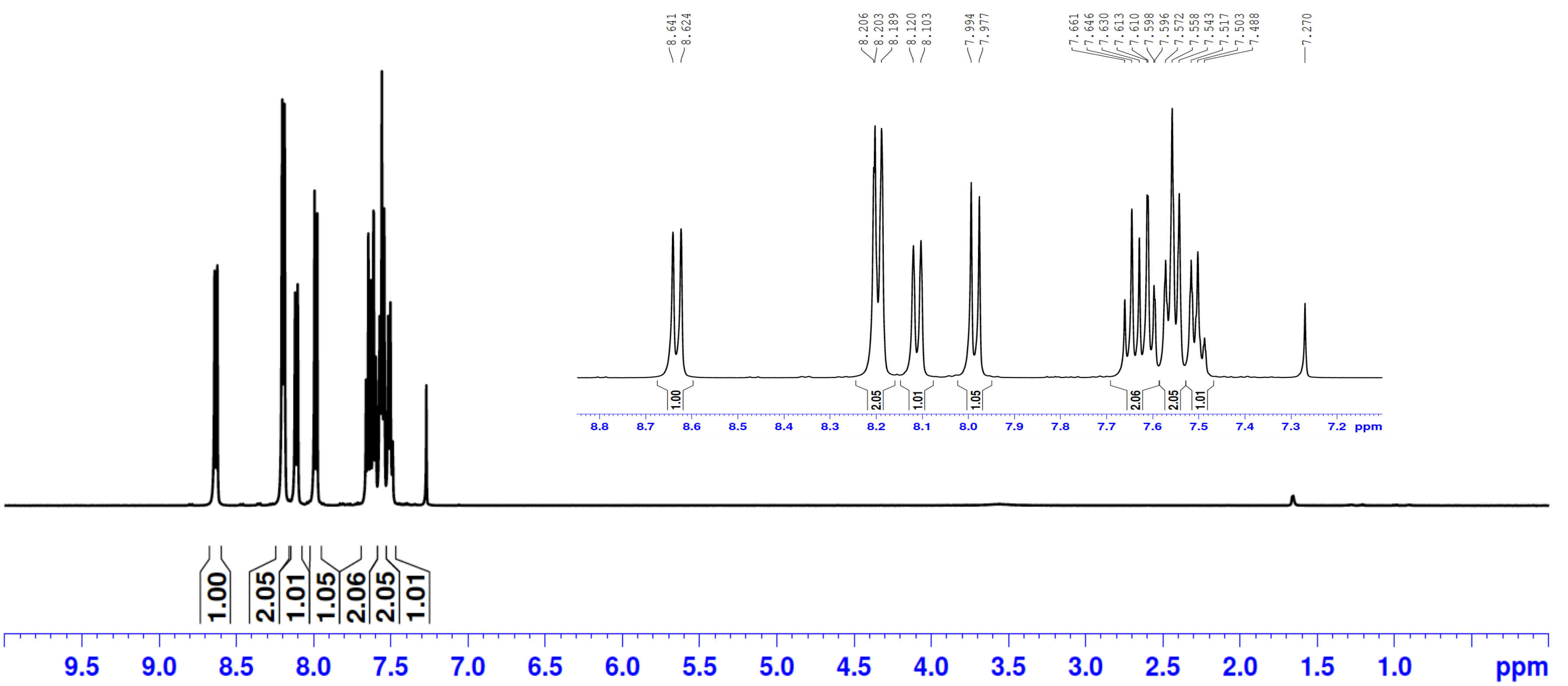

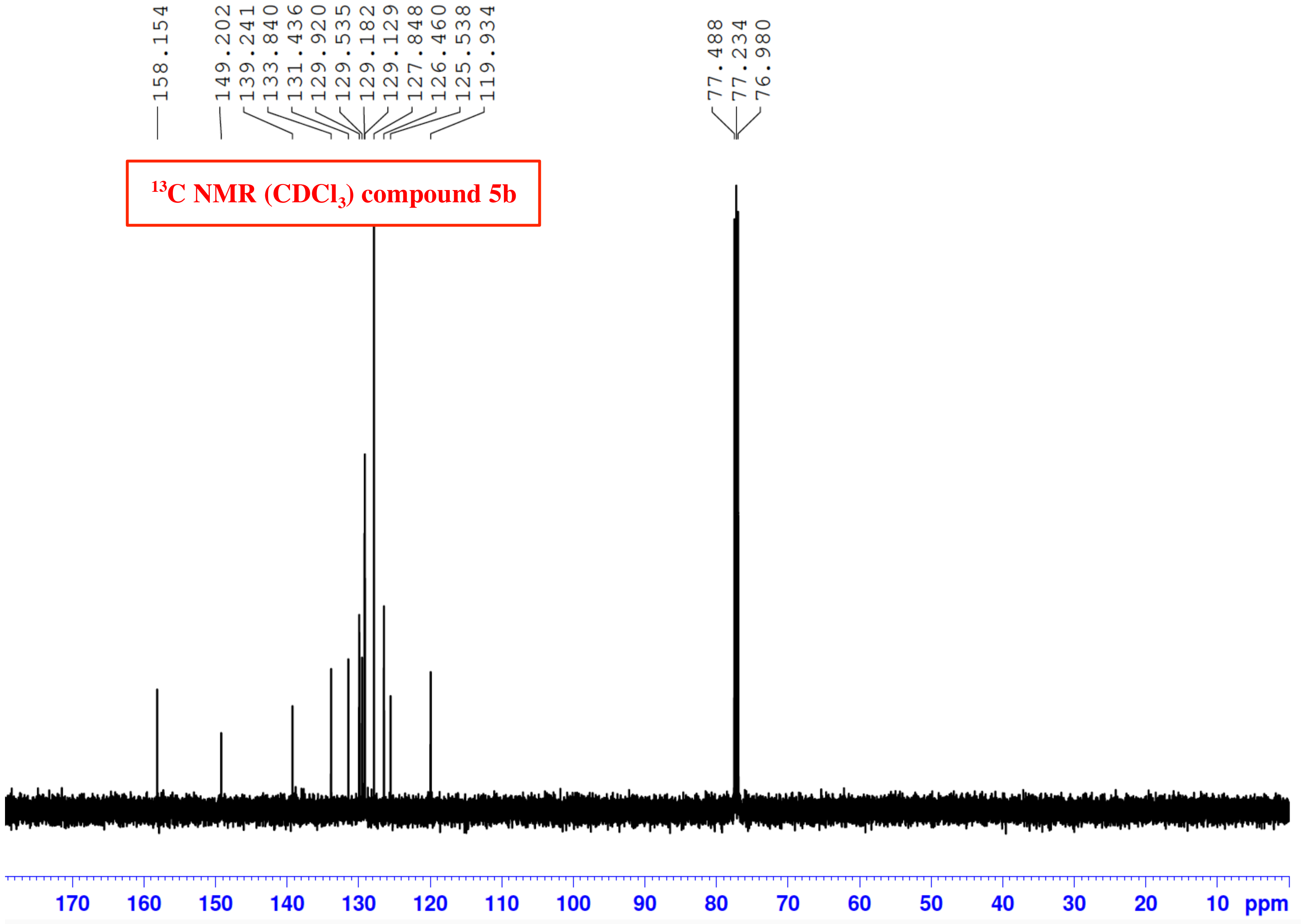
158.154
149.202
139.241
133.840
131.436
129.920
129.535
129.182
129.129
127.848
126.460
125.538
119.934
77.488
77.234
76.980
^{13}C NMR ($CDCl_3$) compound 5b
170
160
150
140
130
120
110
100
90
80
70
60
50
40
30
20
10
ppm

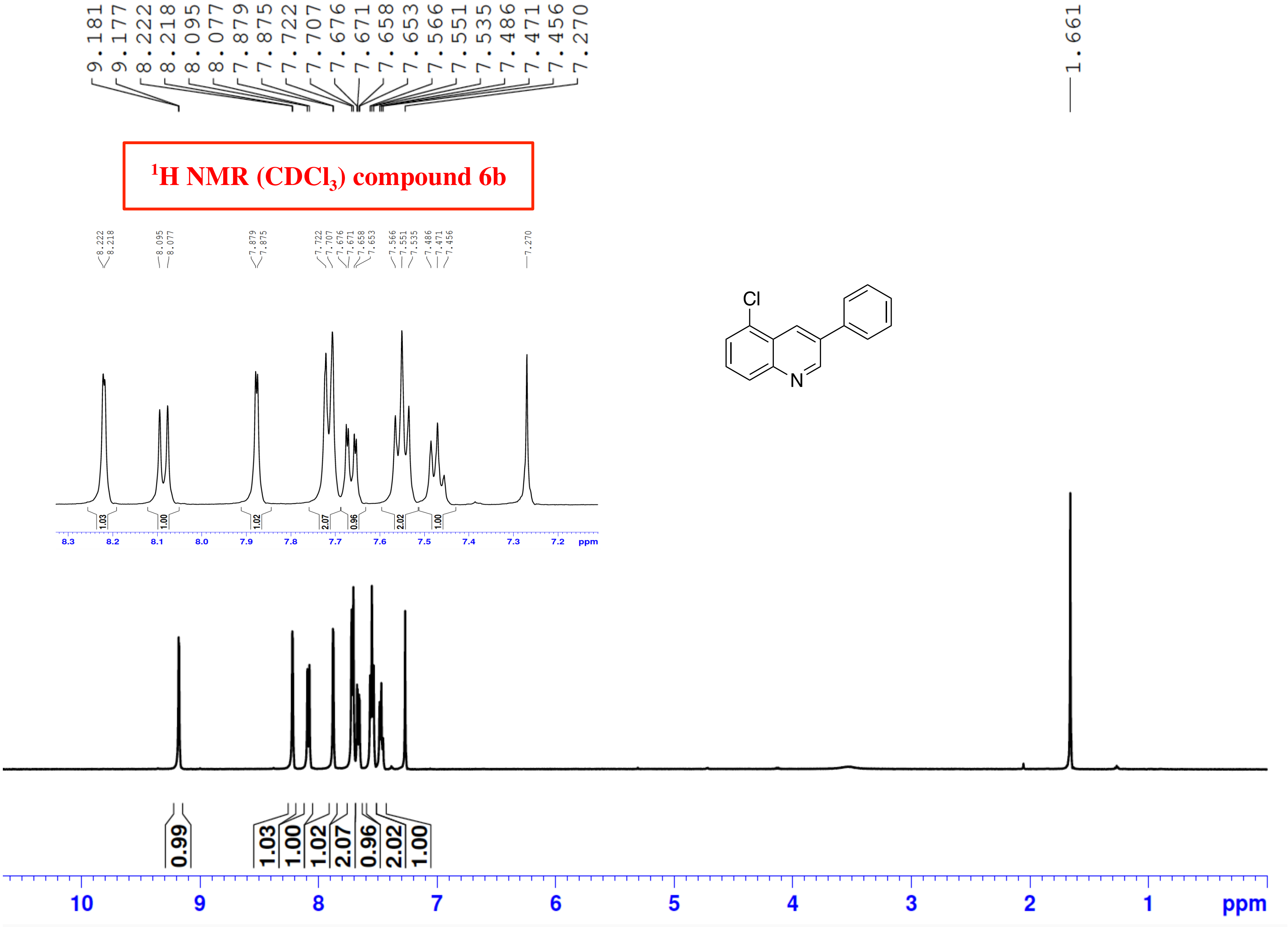
1H NMR (CDCl3) compound 6b
9.181
9.177
8.222
8.218
8.095
8.077
7.879
7.875
7.722
7.707
7.676
7.671
7.658
7.653
7.566
7.551
7.535
7.486
7.471
7.456
7.270
1.661
Cl
N
1.03
1.00
1.02
2.07
0.96
2.02
0.99
ppm

^{13}C NMR ($CDCl_3$) compound 6b

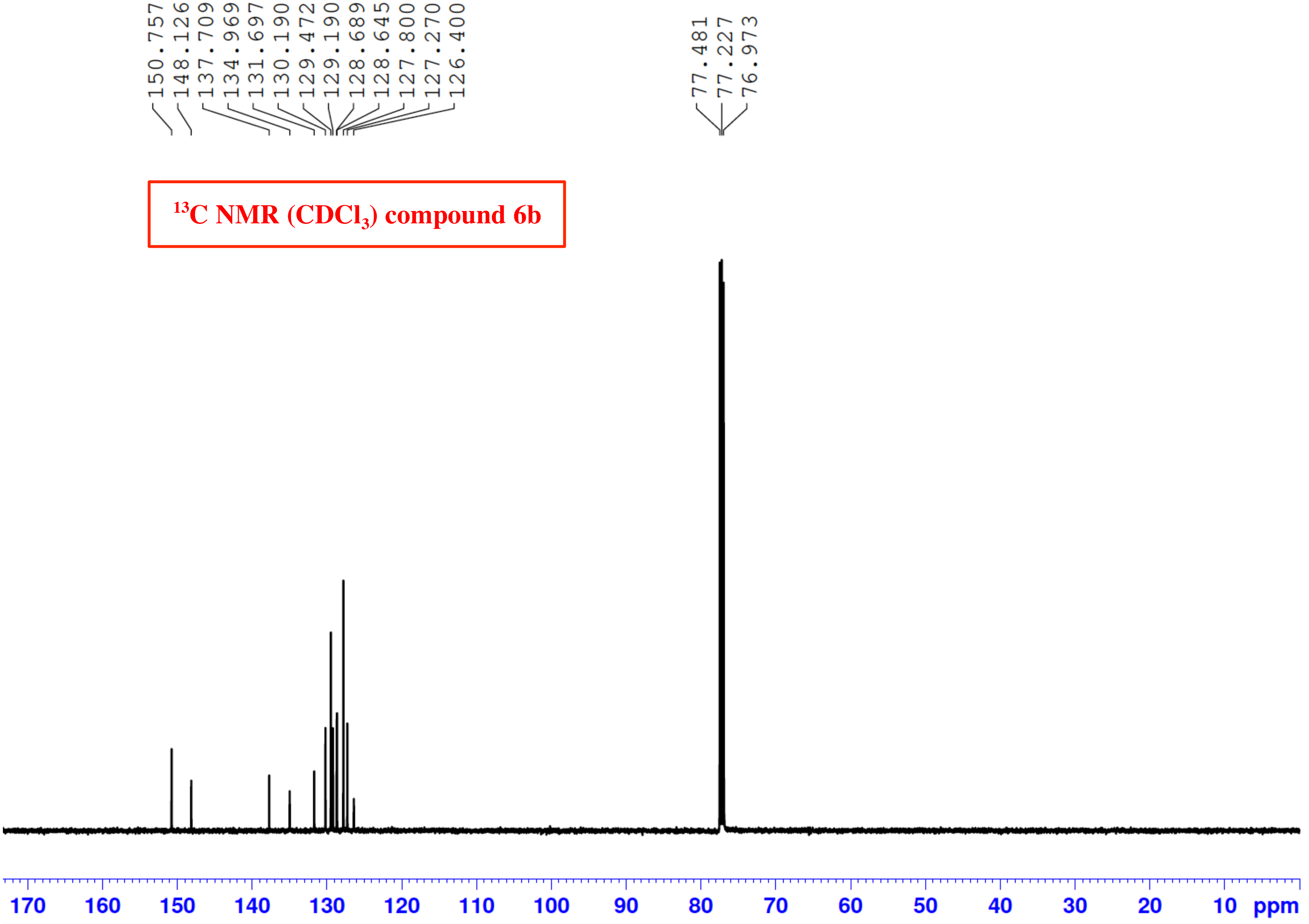

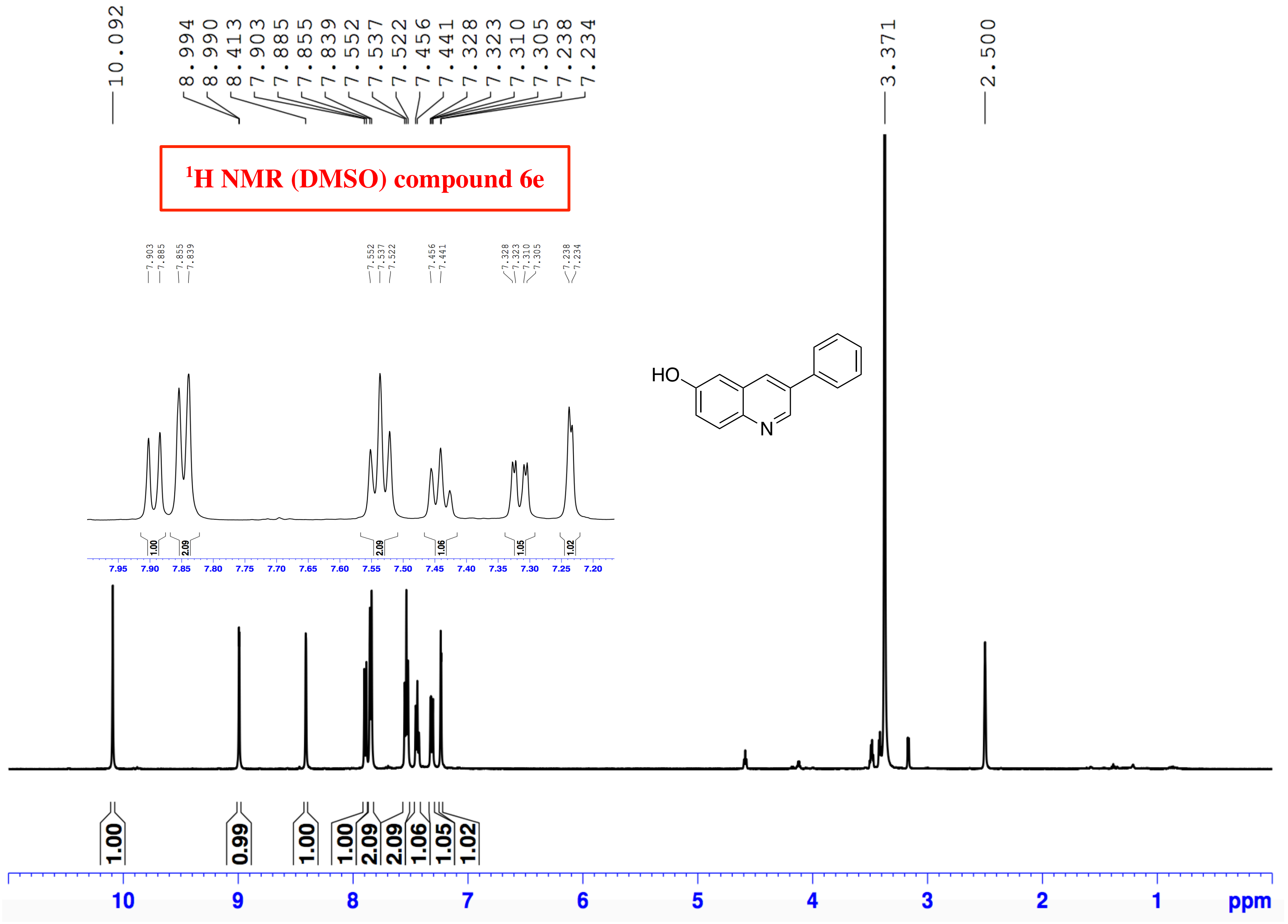
^{1}H NMR (DMSO) compound 6e
HO
N
ppm

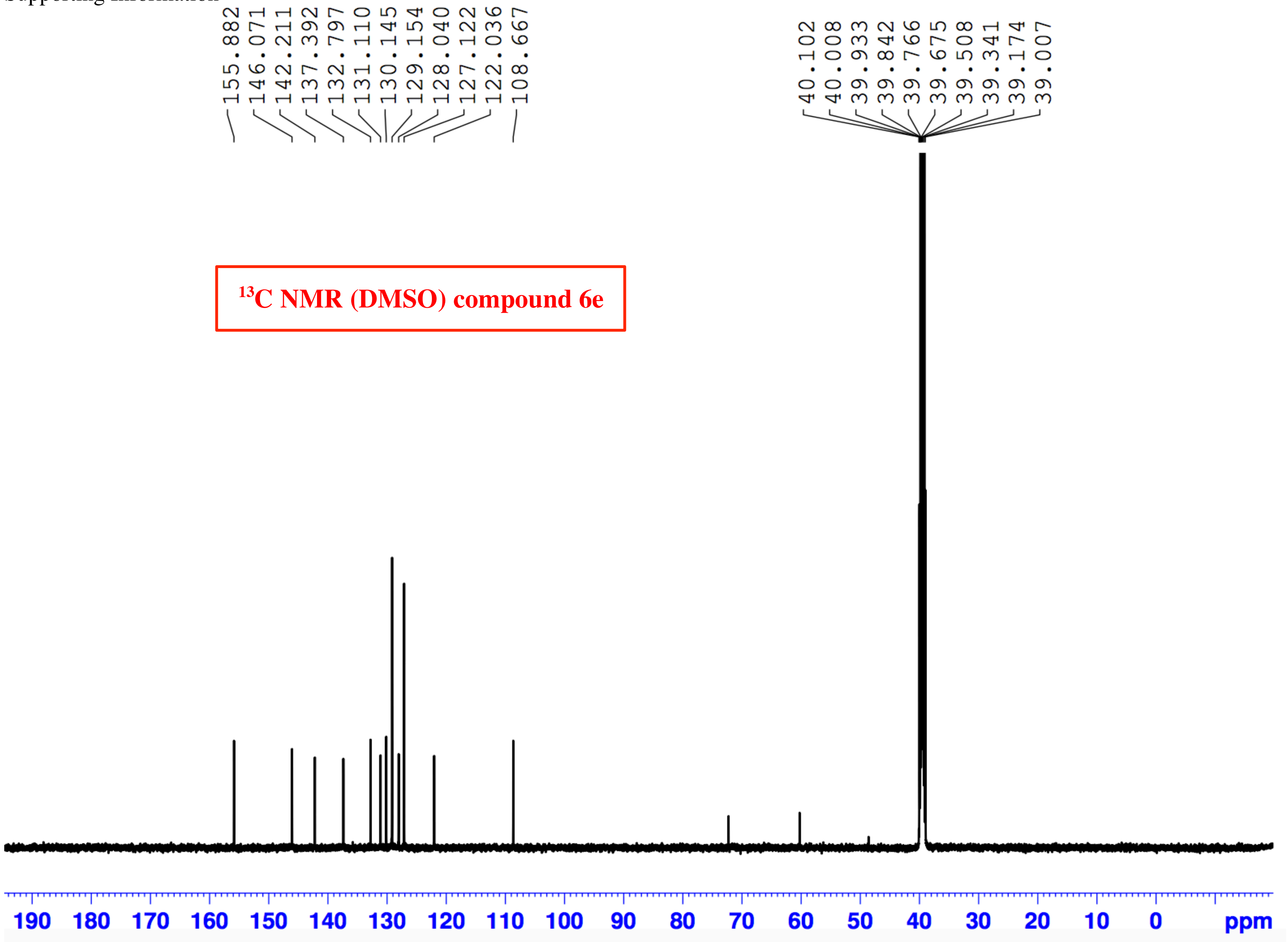
155.882
146.071
142.211
137.392
132.797
131.110
130.145
129.154
128.040
127.122
122.036
108.667
40.102
40.008
39.933
39.842
39.766
39.675
39.508
39.341
39.174
39.007
^{13}C NMR (DMSO) compound 6e
190 180 170 160 150 140 130 120 110 100 90 80 70 60 50 40 30 20 10 0 ppm

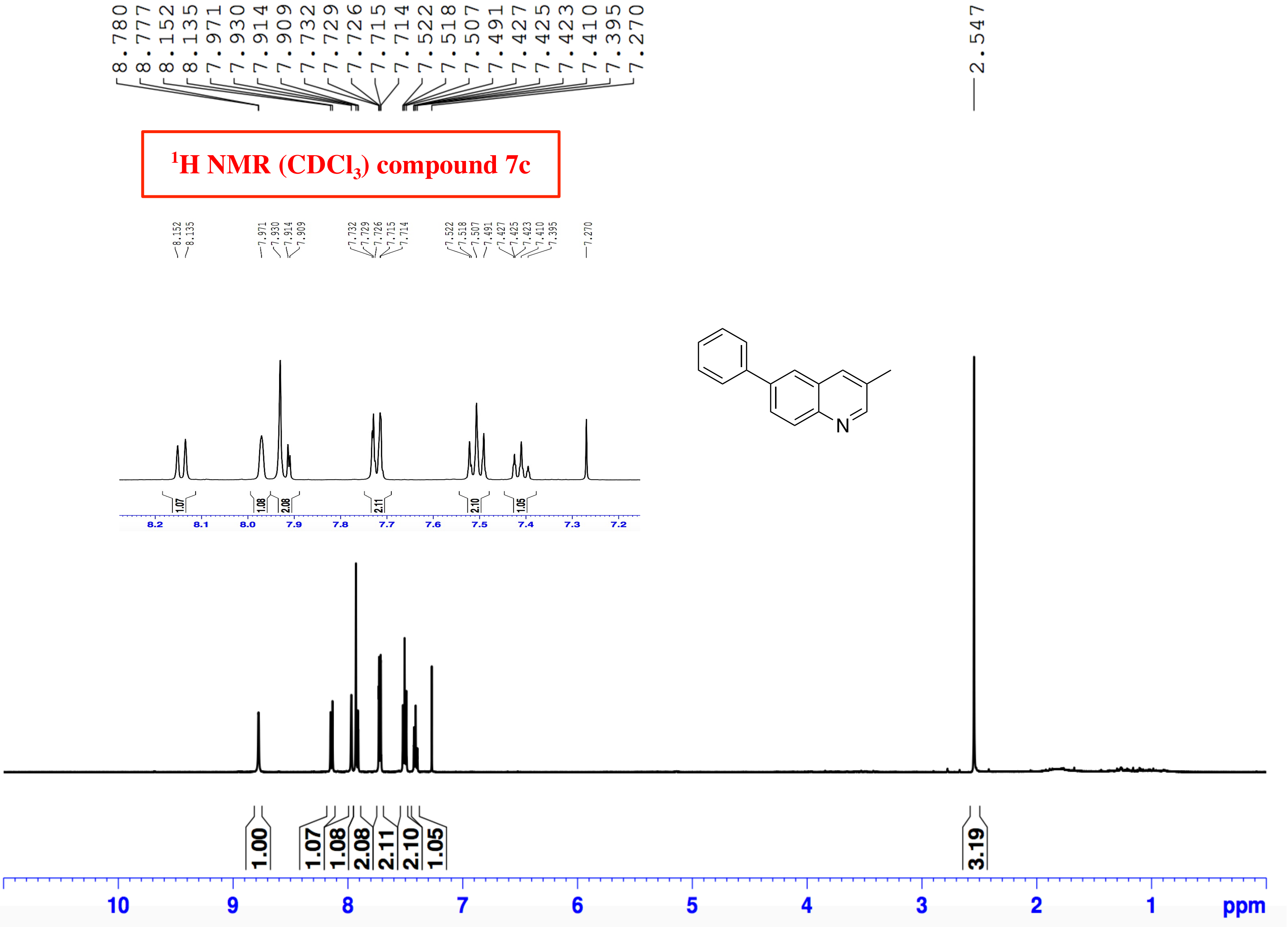
8.780
8.777
8.152
8.135
7.971
7.930
7.914
7.909
7.732
7.729
7.726
7.715
7.714
7.522
7.518
7.507
7.491
7.427
7.425
7.423
7.410
7.395
7.270
2.547
¹H NMR (CDCl₃) compound 7c
N
1.07
1.08
2.08
2.11
2.10
1.05
1.00
3.19
ppm

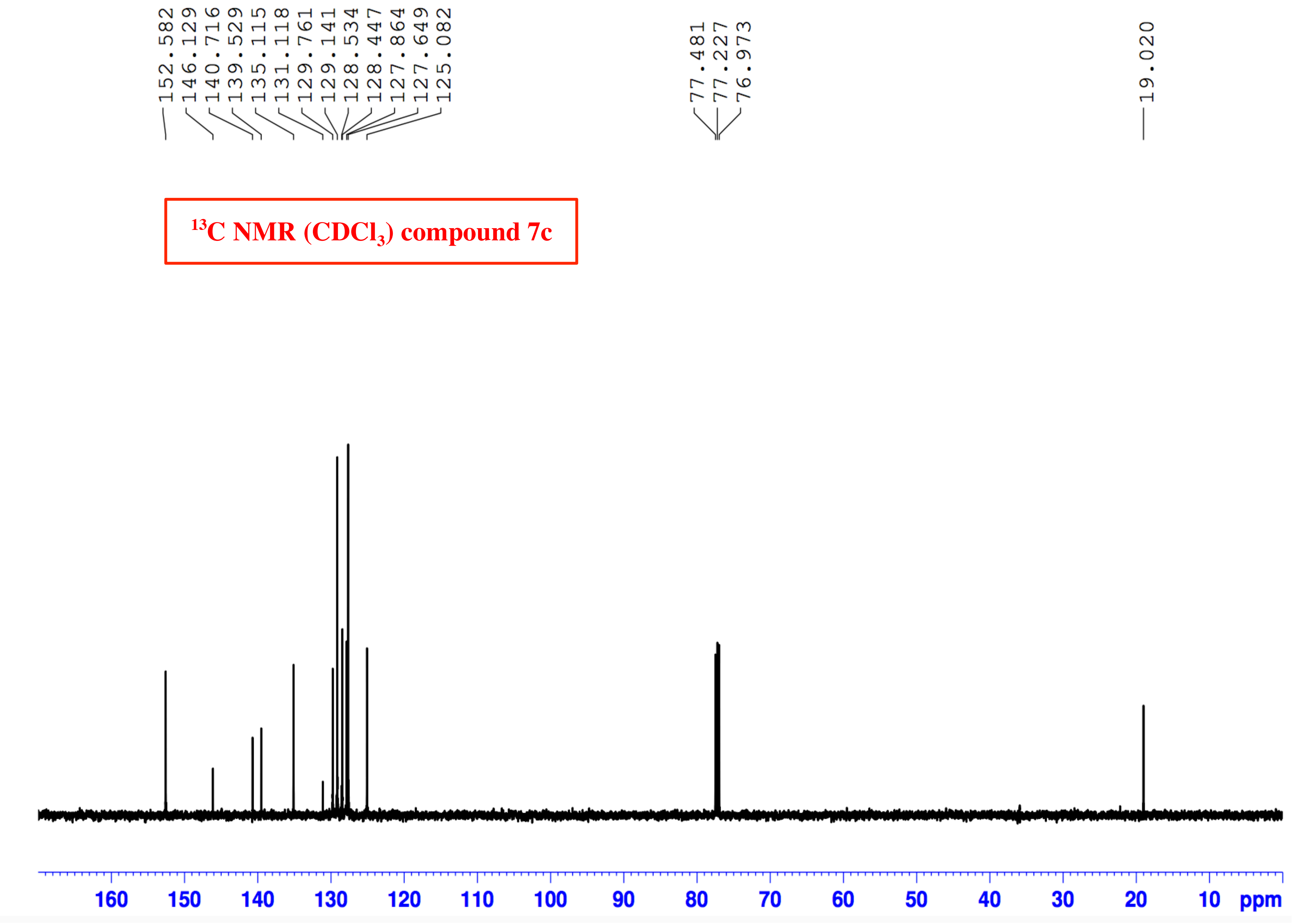
152.582
146.129
140.716
139.529
135.115
131.118
129.761
129.141
128.534
128.447
127.864
127.649
125.082
77.481
77.227
76.973
19.020
13C NMR (CDCl3) compound 7c
160
150
140
130
120
110
100
90
80
70
60
50
40
30
20
10
ppm

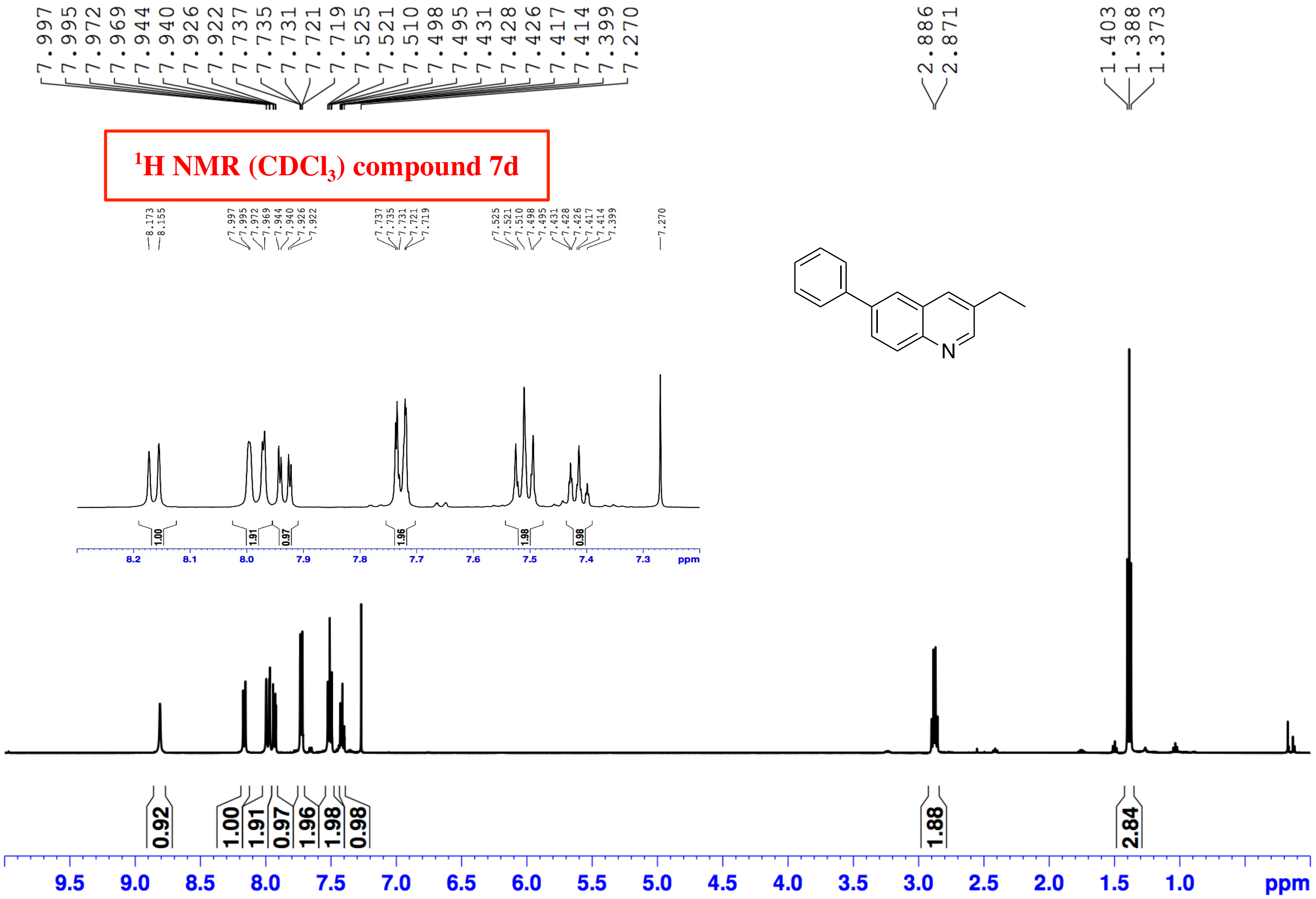

^{1}H NMR (CDCl3) compound 7d
7.997 7.995 7.972 7.969 7.944 7.940 7.926 7.922 7.737 7.735 7.731 7.721 7.719 7.525 7.521 7.510 7.498 7.495 7.431 7.428 7.426 7.417 7.414 7.399 7.270
2.886 2.871
1.403 1.388 1.373
8.173 8.155
N
1.00 1.91 0.97 1.96 1.98 0.98
8.2 8.1 8.0 7.9 7.8 7.7 7.6 7.5 7.4 7.3 ppm
0.92 1.00 1.91 0.97 1.96 1.98 0.98 1.88 2.84
9.5 9.0 8.5 8.0 7.5 7.0 6.5 6.0 5.5 5.0 4.5 4.0 3.5 3.0 2.5 2.0 1.5 1.0 ppm

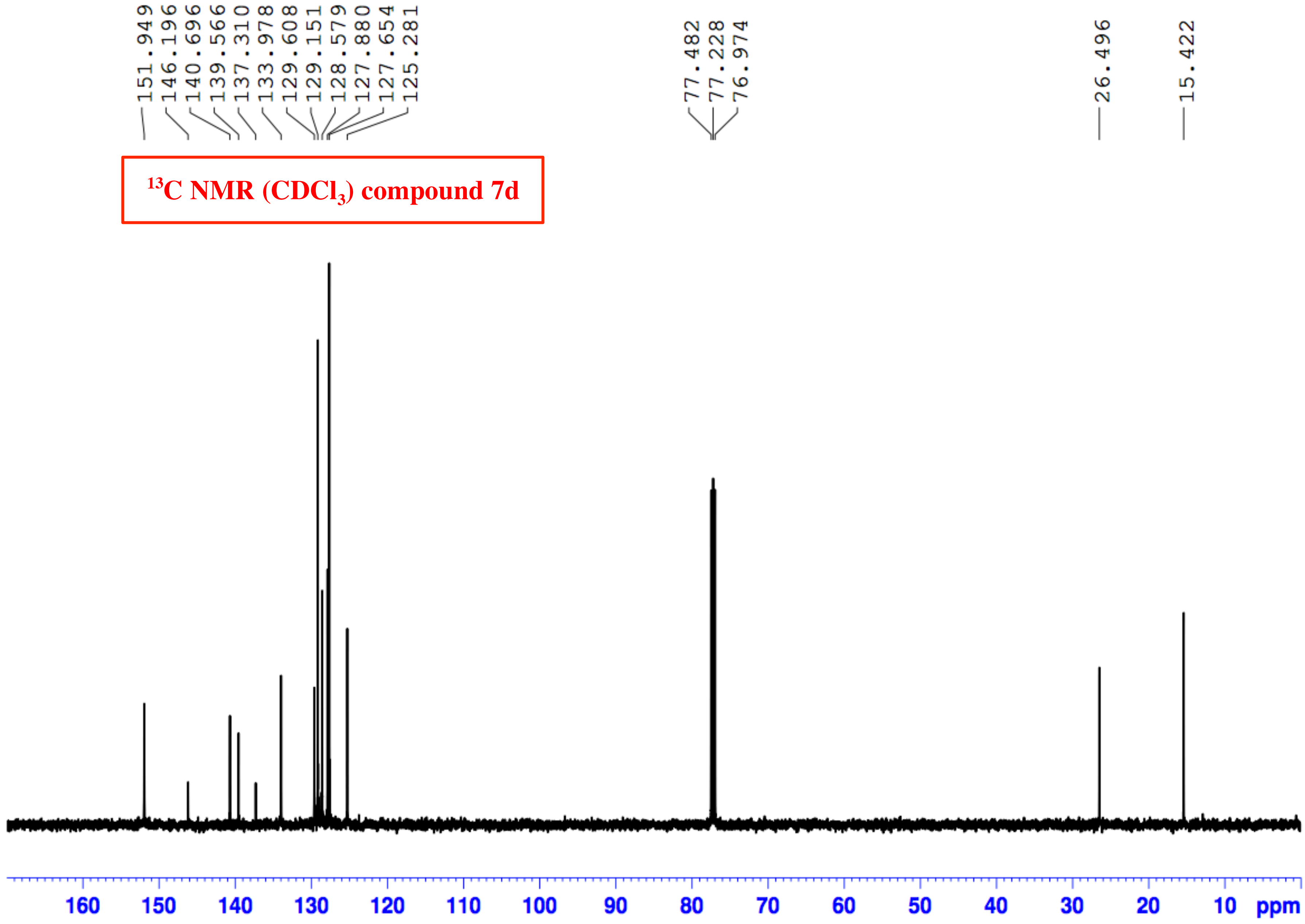
151.949
146.196
140.696
139.566
137.310
133.978
129.608
129.151
128.579
127.880
127.654
125.281
77.482
77.228
76.974
26.496
15.422
^{13}C NMR ($CDCl_3$) compound 7d
160
150
140
130
120
110
100
90
80
70
60
50
40
30
20
10
ppm

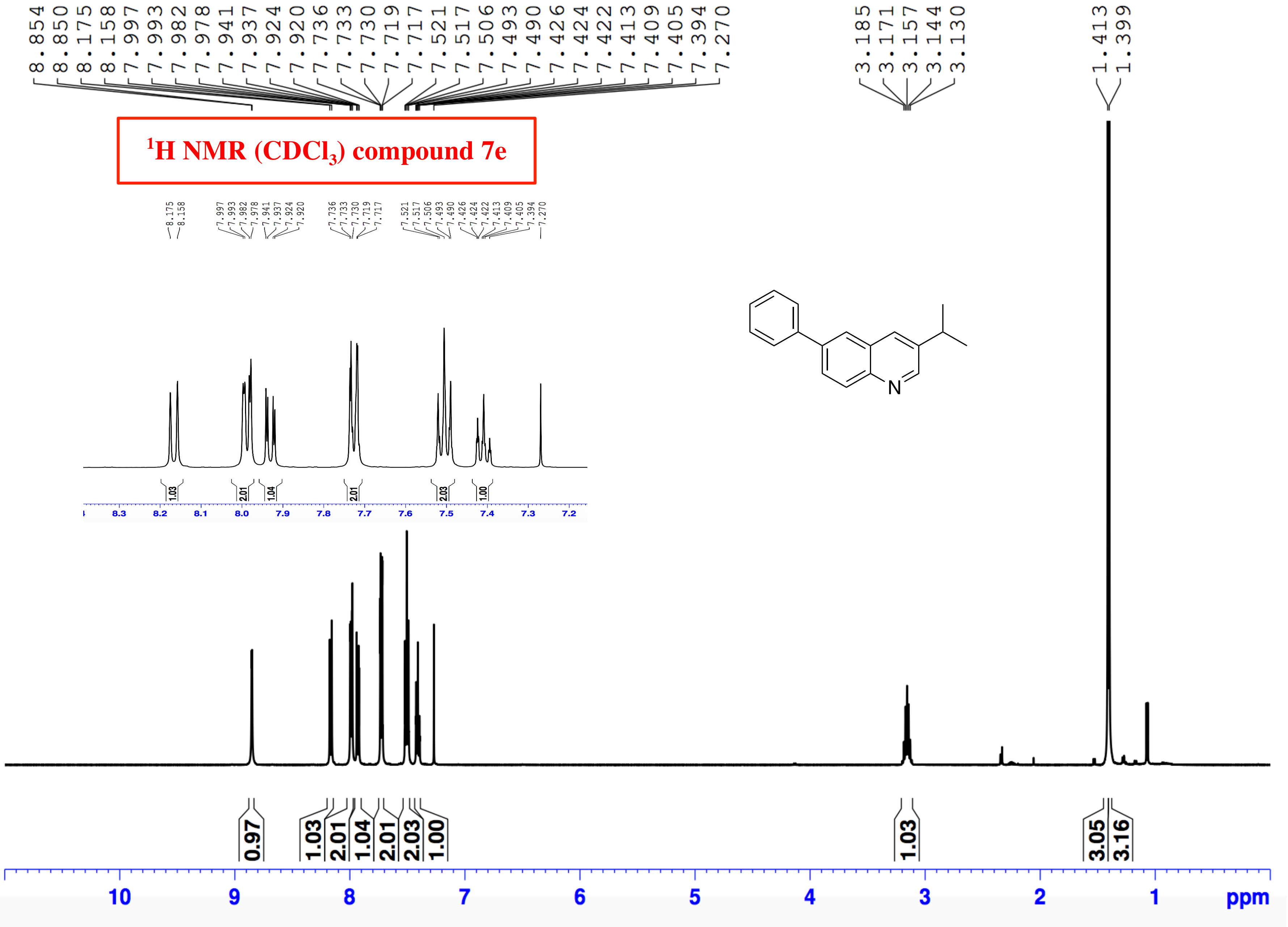
8.854
8.850
8.175
8.158
7.997
7.993
7.982
7.978
7.941
7.937
7.924
7.920
7.736
7.733
7.730
7.719
7.717
7.521
7.517
7.506
7.493
7.490
7.426
7.424
7.422
7.413
7.409
7.405
7.394
7.270
3.185
3.171
3.157
3.144
3.130
1.413
1.399
1H NMR (CDCl3) compound 7e
N
0.97
1.03
2.01
1.04
2.01
2.03
1.00
1.03
3.05
3.16
10
9
8
7
6
5
4
3
2
1
ppm

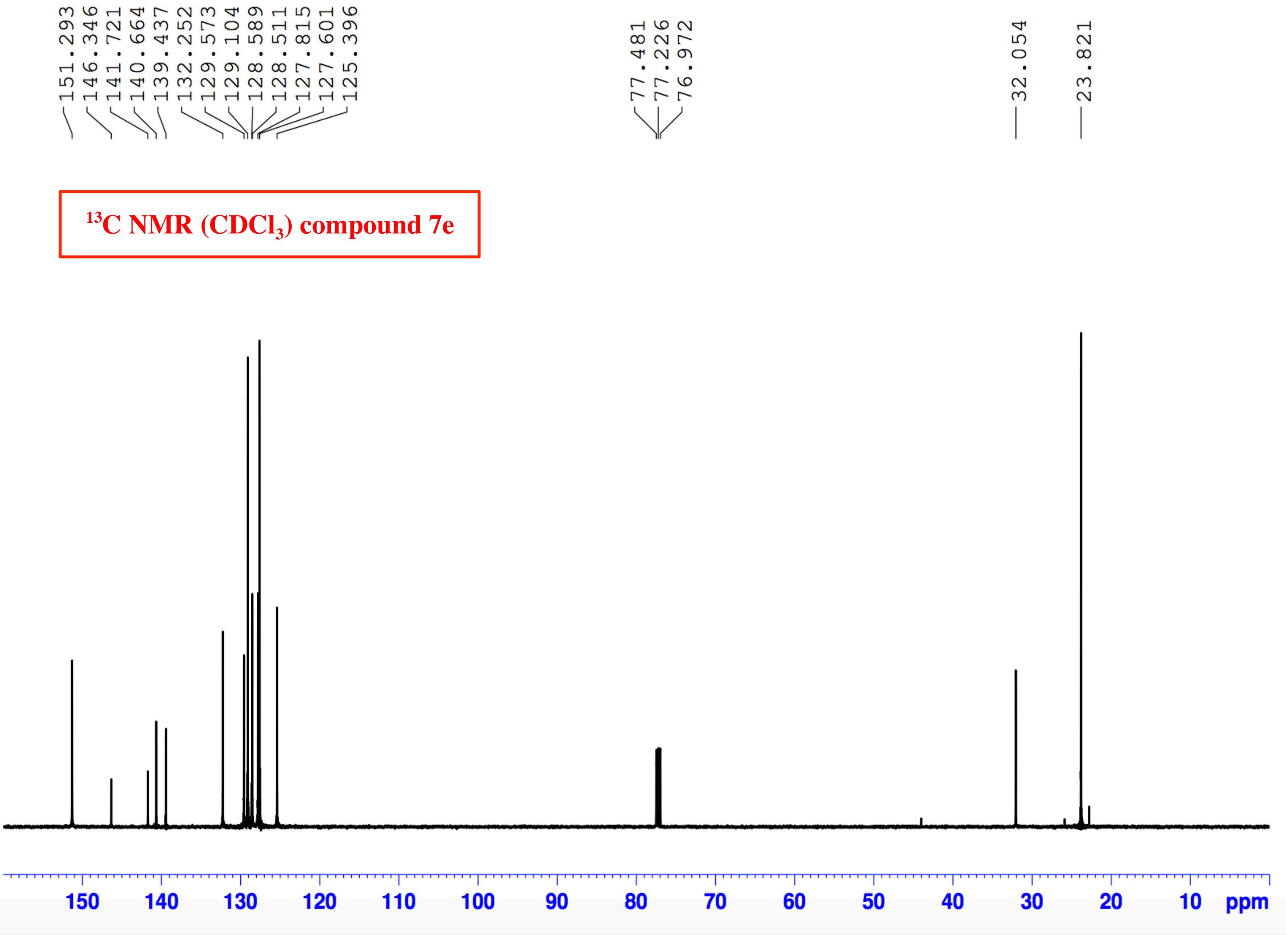
151.293
146.346
141.721
140.664
139.437
132.252
129.573
129.104
128.589
128.511
127.815
127.601
125.396
77.481
77.226
76.972
32.054
23.821
^{13}C NMR ($CDCl_3$) compound 7e
150
140
130
120
110
100
90
80
70
60
50
40
30
20
10
ppm

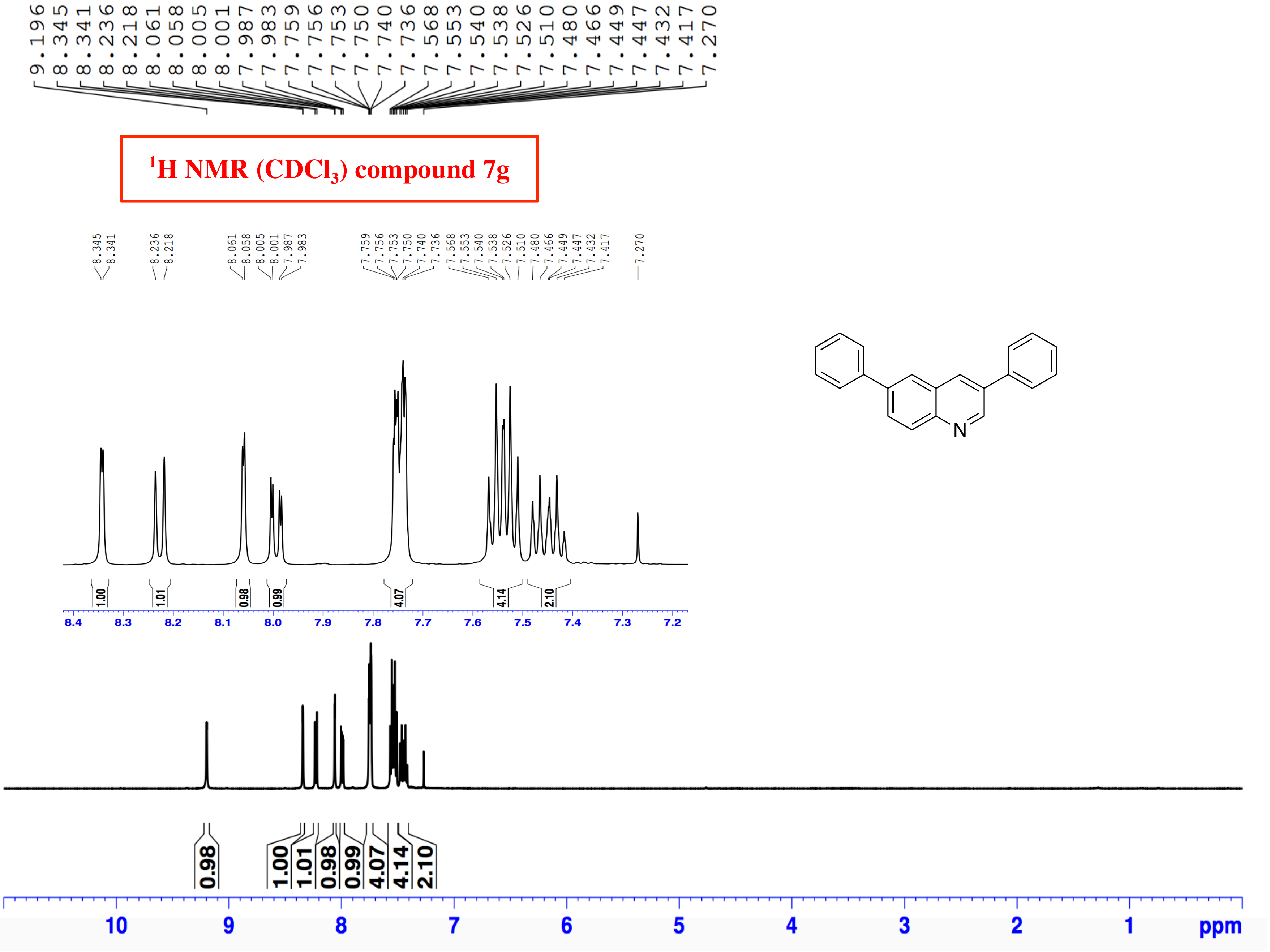
9.196 8.345 8.341 8.236 8.218 8.061 8.058 8.005 8.001 7.987 7.983 7.759 7.756 7.753 7.750 7.740 7.736 7.568 7.553 7.540 7.538 7.526 7.510 7.480 7.466 7.449 7.447 7.432 7.417 7.270
1H NMR (CDCl3) compound 7g
1.00 1.01 0.98 0.99 4.07 4.14 2.10
N
0.98 1.00 1.01 0.98 0.99 4.07 4.14 2.10
ppm

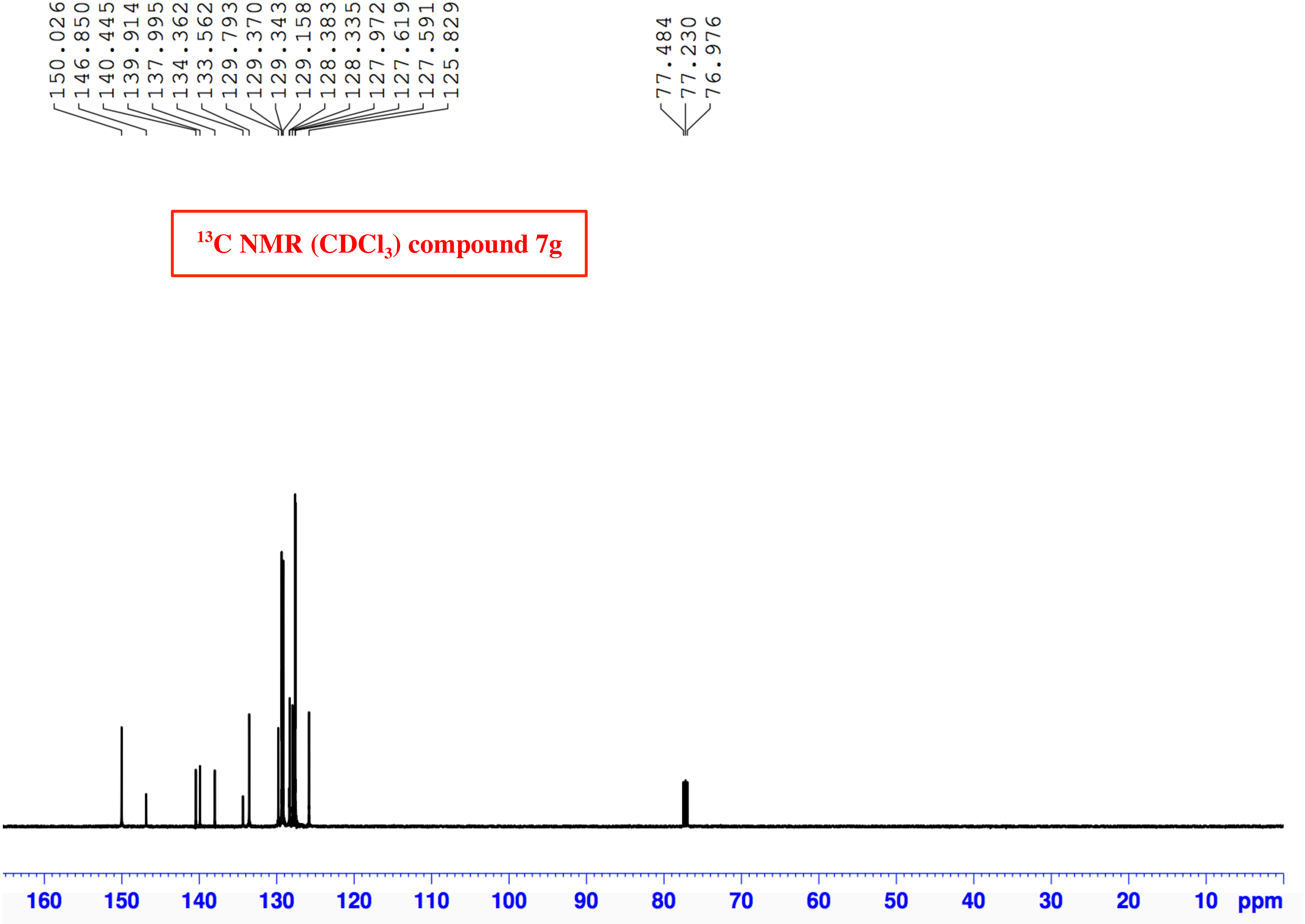
150.026
146.850
140.445
139.914
137.995
134.362
133.562
129.793
129.370
129.343
129.158
128.383
128.335
127.972
127.619
127.591
125.829
77.484
77.230
76.976
^{13}C NMR ($CDCl_3$) compound 7g
160
150
140
130
120
110
100
90
80
70
60
50
40
30
20
10
ppm

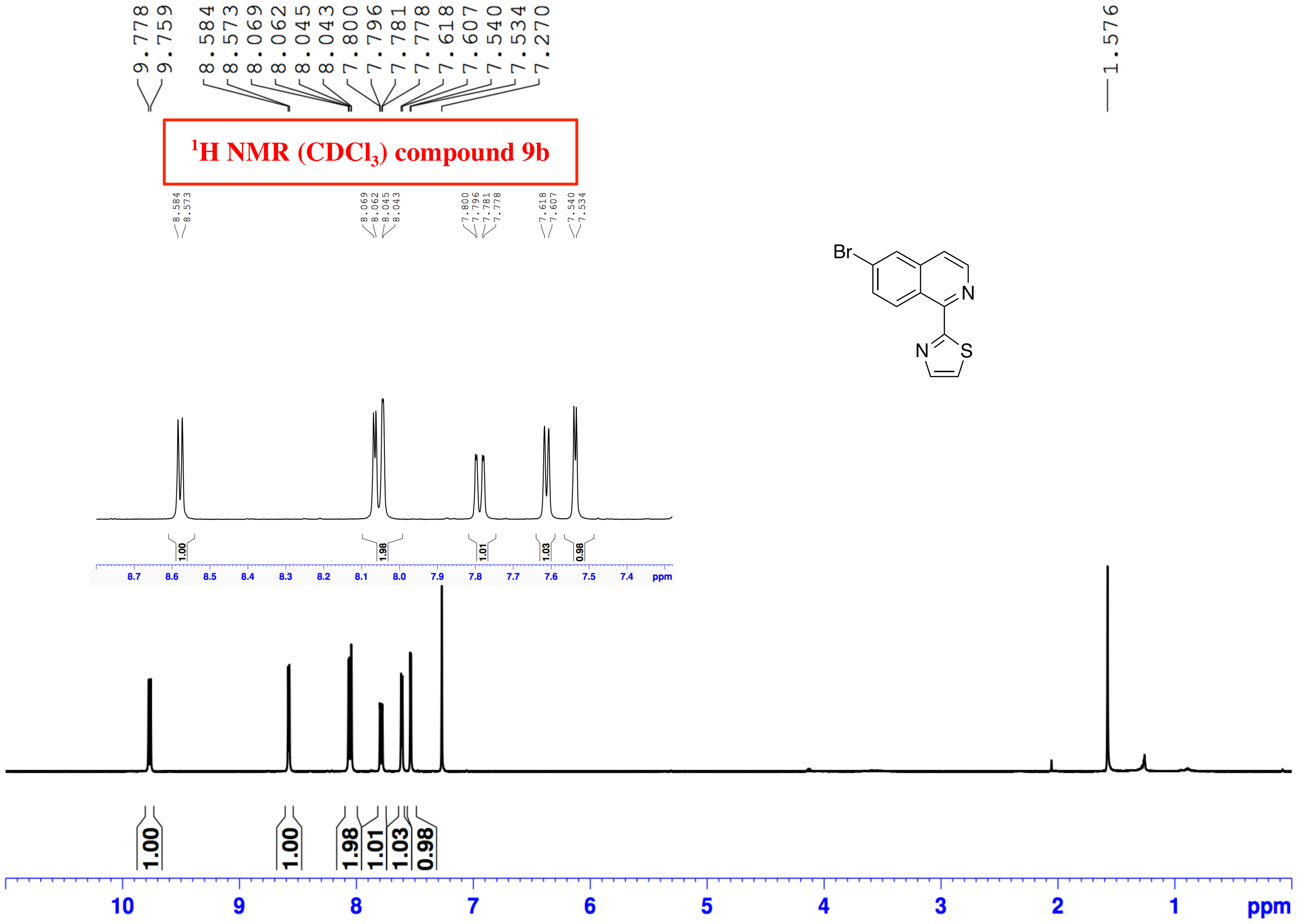
9.778
9.759
8.584
8.573
8.069
8.062
8.045
8.043
7.800
7.796
7.781
7.778
7.618
7.607
7.540
7.534
7.270
1.576
1H NMR (CDCl3) compound 9b
Br
N
N
S
1.00
1.98
1.01
1.03
0.98
8.7
8.6
8.5
8.4
8.3
8.2
8.1
8.0
7.9
7.8
7.7
7.6
7.5
7.4
ppm
1.00
1.00
1.98
1.01
1.03
0.98
10
9
8
7
6
5
4
3
2
1
ppm

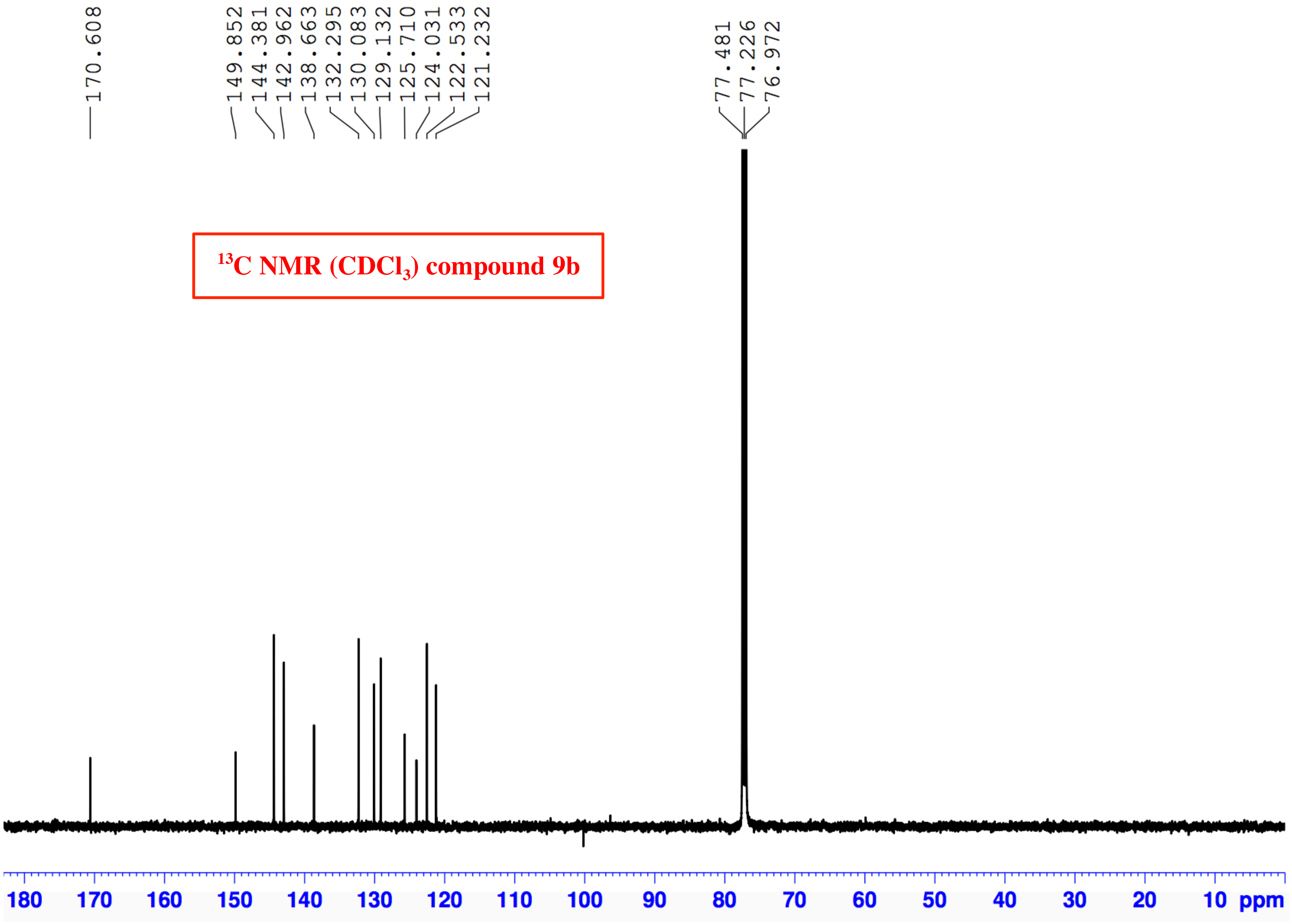
170.608
149.852
144.381
142.962
138.663
132.295
130.083
129.132
125.710
124.031
122.533
121.232
77.481
77.226
76.972
13C NMR (CDCl3) compound 9b
180 170 160 150 140 130 120 110 100 90 80 70 60 50 40 30 20 10 ppm

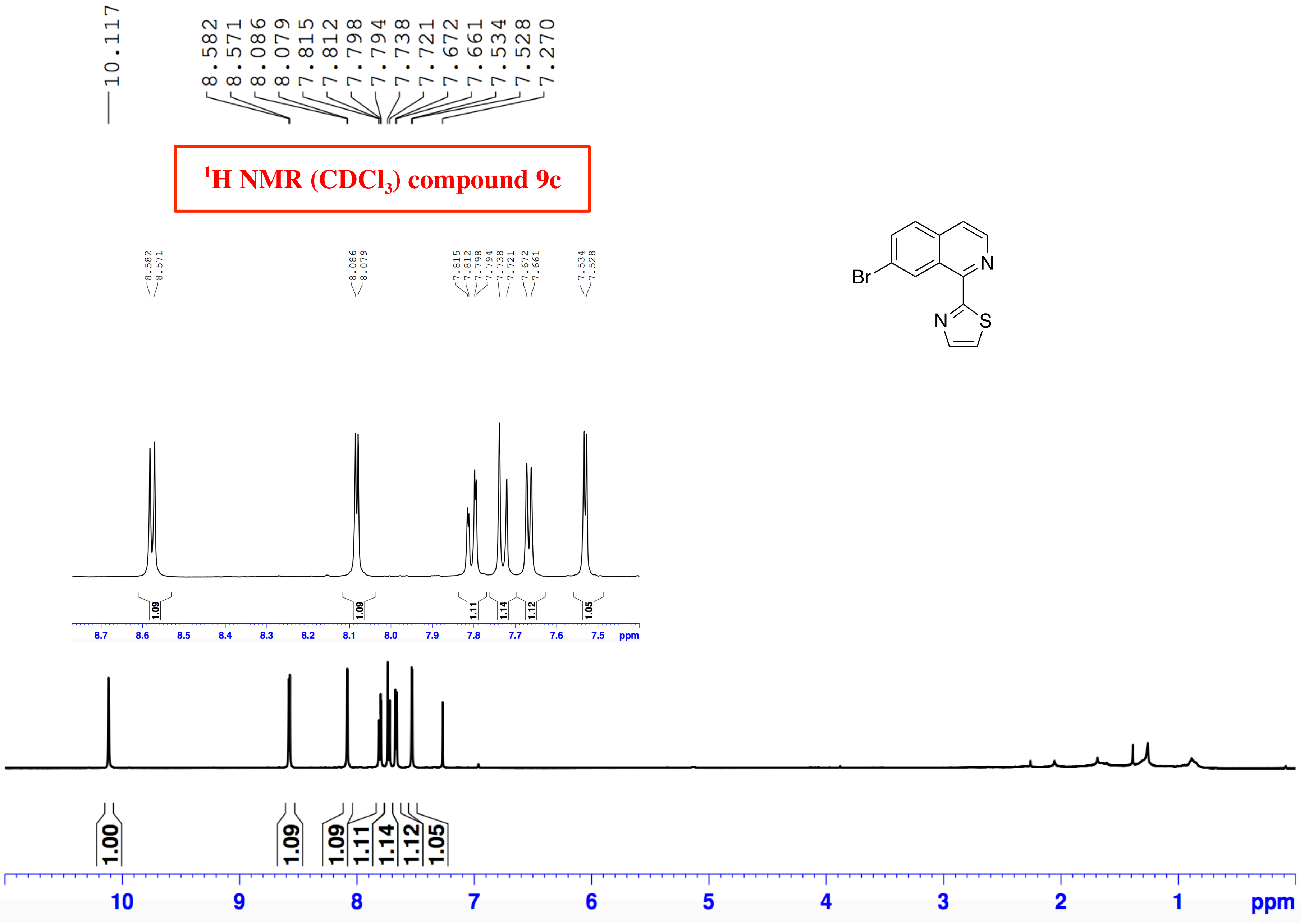
^{1}H NMR (CDCl3) compound 9c
10.117
8.582
8.571
8.086
8.079
7.815
7.812
7.798
7.794
7.738
7.721
7.672
7.661
7.534
7.528
7.270
1.00
1.09
1.09
1.11
1.14
1.12
1.05
ppm
Br
N
N
S

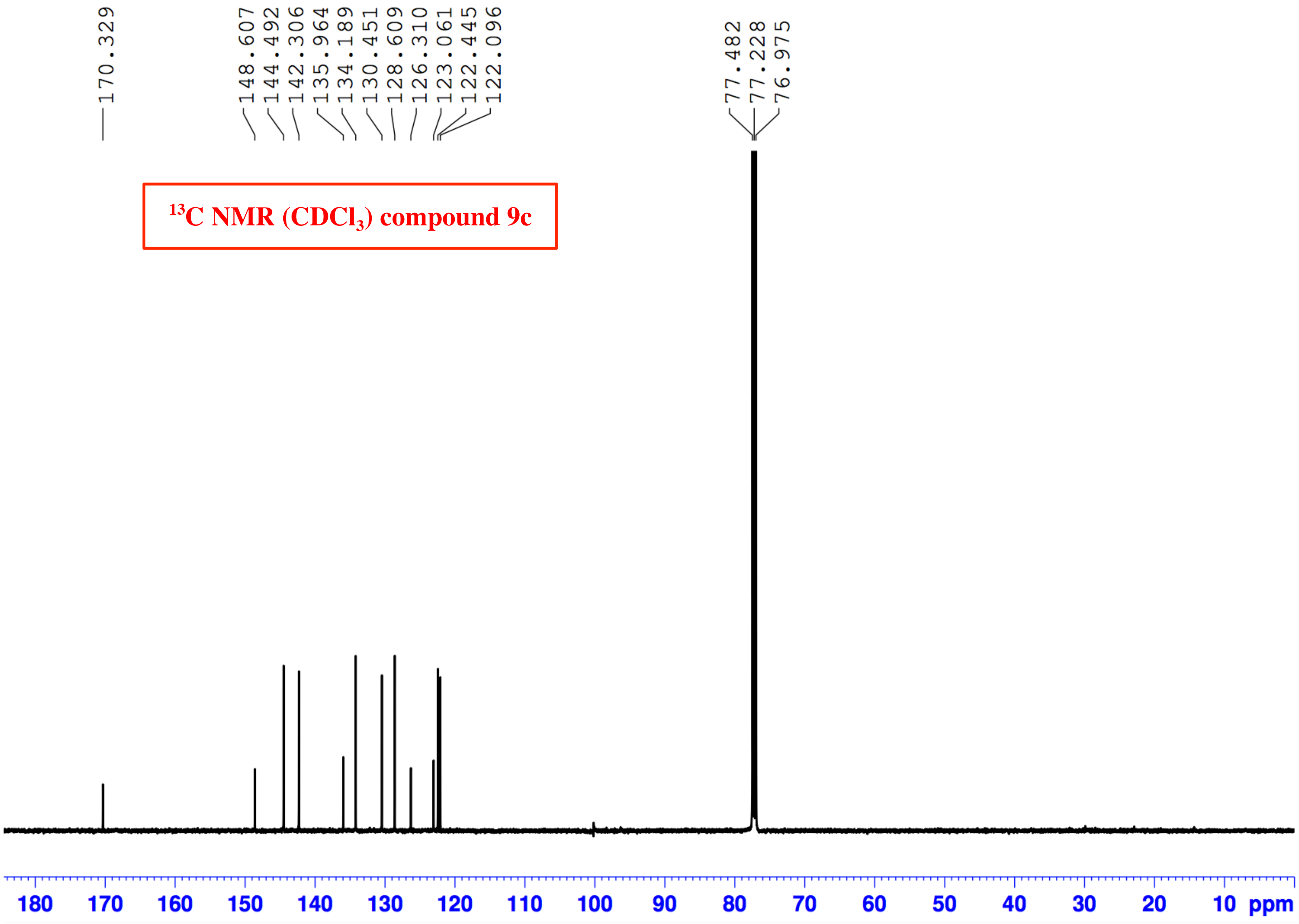
170.329
148.607
144.492
142.306
135.964
134.189
130.451
128.609
126.310
123.061
122.445
122.096
77.482
77.228
76.975
13C NMR (CDCl3) compound 9c
180
170
160
150
140
130
120
110
100
90
80
70
60
50
40
30
20
10
ppm

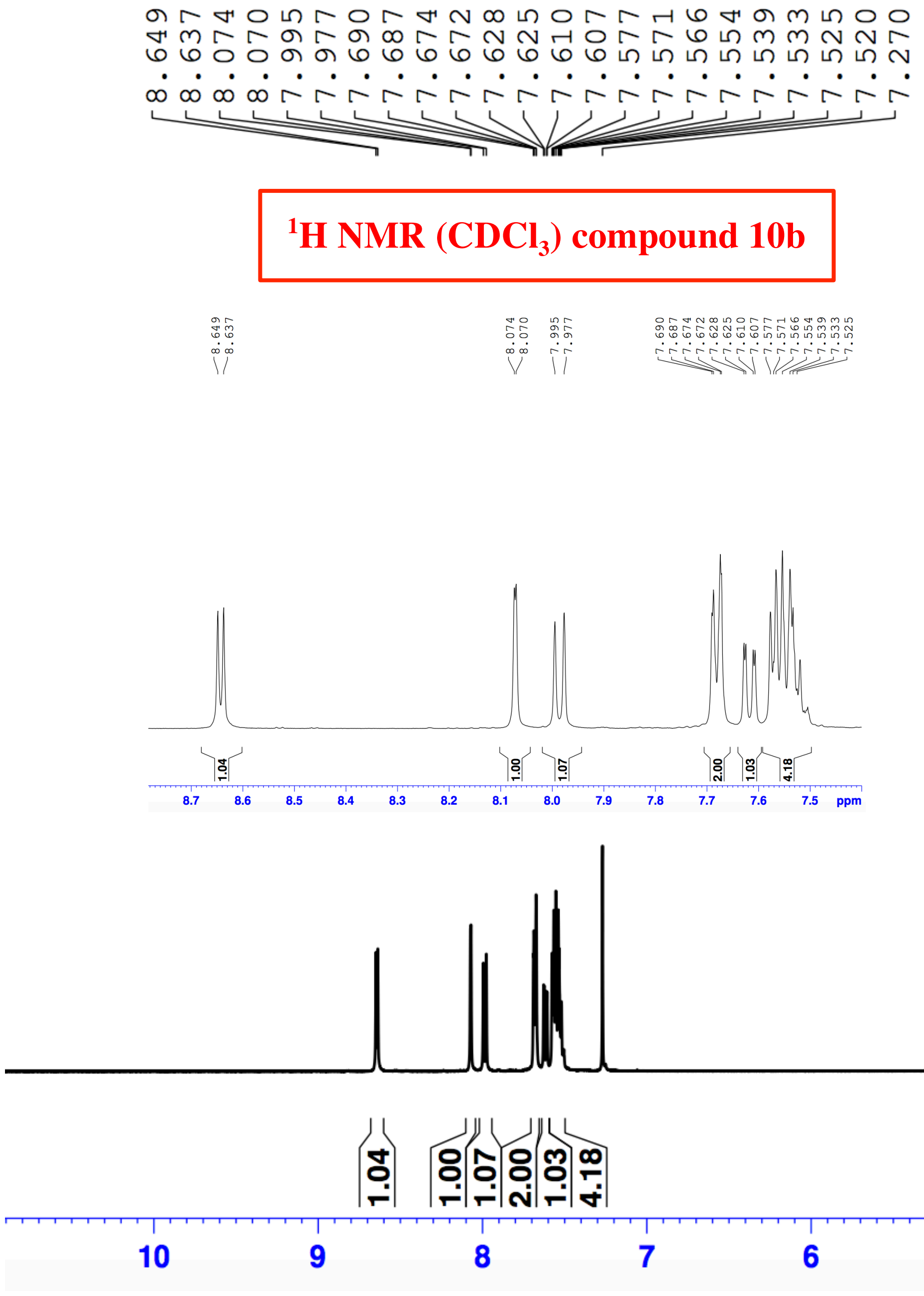
^{1}H NMR (CDCl3) compound 10b
8.649
8.637
8.074
8.070
7.995
7.977
7.690
7.687
7.674
7.672
7.628
7.625
7.610
7.607
7.577
7.571
7.566
7.554
7.539
7.533
7.525
7.520
7.270
1.04
1.00
1.07
2.00
1.03
4.18
ppm

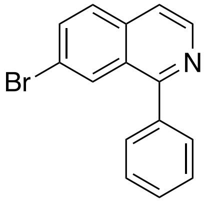
Br
N

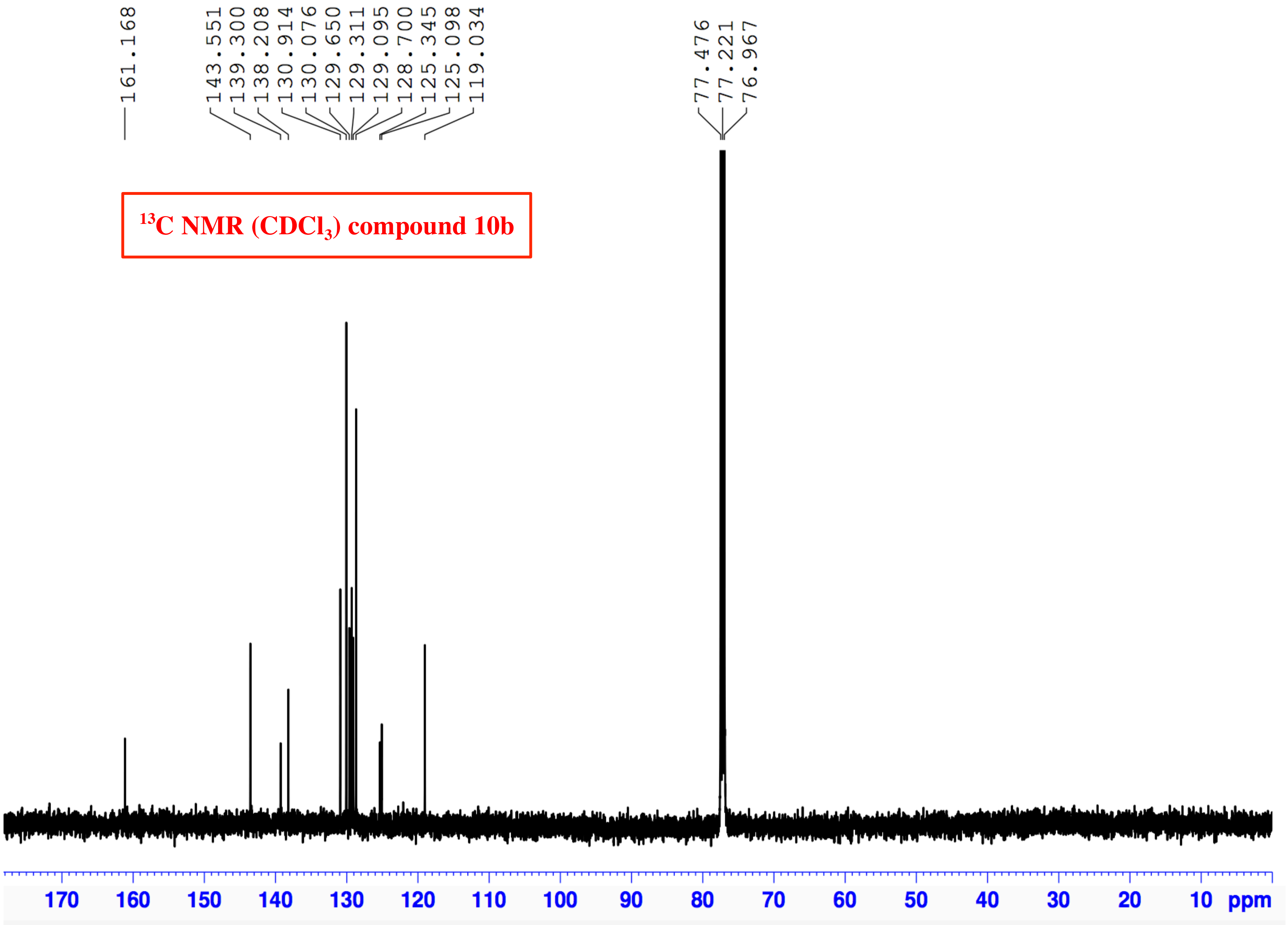
161.168
143.551
139.300
138.208
130.914
130.076
129.650
129.311
129.095
128.700
125.345
125.098
119.034
77.476
77.221
76.967
^{13}C NMR ($CDCl_3$) compound 10b
170
160
150
140
130
120
110
100
90
80
70
60
50
40
30
20
10
ppm
